# The Natterin Proteins Diversity: A Review on Phylogeny, Structure, and Immune Function

**DOI:** 10.3390/toxins13080538

**Published:** 2021-07-31

**Authors:** Carla Lima, Geonildo Rodrigo Disner, Maria Alice Pimentel Falcão, Ana Carolina Seni-Silva, Adolfo Luis Almeida Maleski, Milena Marcolino Souza, Mayara Cristina Reis Tonello, Monica Lopes-Ferreira

**Affiliations:** 1 Immunoregulation Unit of the Laboratory of Applied Toxinology (CeTICs/FAPESP), Butantan Institute, Vital Brasil Avenue, 1500 Butantan, São Paulo 05503-900, Brazil; disner.rodrigo@gmail.com (G.R.D.); maria.falcao@esib.butantan.gov.br (M.A.P.F.); ana.seni@esib.butantan.gov.br (A.C.S.-S.); adolfo.maleski@esib.butantan.gov.br (A.L.A.M.); milenamarcolino.mm@gmail.com (M.M.S.); maychreis@gmail.com (M.C.R.T.); monica.lopesferreira@butantan.gov.br (M.L.-F.); 2 Post-Graduation Program of Toxinology, Butantan Institute, São Paulo 05503-900, Brazil

**Keywords:** *Thalassophryne nattereri*, Natterin, aerolysin, protein evolution, immune function, bioinformatics

## Abstract

Since the first record of the five founder members of the group of Natterin proteins in the venom of the medically significant fish *Thalassophryne nattereri*, new sequences have been identified in other species. In this work, we performed a detailed screening using available genome databases across a wide range of species to identify sequence members of the Natterin group, sequence similarities, conserved domains, and evolutionary relationships. The high-throughput tools have enabled us to dramatically expand the number of members within this group of proteins, which has a remote origin (around 400 million years ago) and is spread across Eukarya organisms, even in plants and primitive Agnathans jawless fish. Overall, the survey resulted in 331 species presenting Natterin-like proteins, mainly fish, and 859 putative genes. Besides fish, the groups with more species included in our analysis were insects and birds. The number and variety of annotations increased the knowledge of the obtained sequences in detail, such as the conserved motif AGIP in the pore-forming loop involved in the transmembrane barrel insertion, allowing us to classify them as important constituents of the innate immune defense system as effector molecules activating immune cells by interacting with conserved intracellular signaling mechanisms in the hosts.

## 1. Introduction

The Natterin proteins were first revealed in the venom of the medically significant Brazilian toadfish *Thalassophryne nattereri* (V*Tn*) in five orthologs named Natterin (1–4, and -P) [1]. They were identified as being responsible for the main toxic non-lethal effects of the V*Tn*, such as local edema and excruciating pain, that evolved to necrosis [2,3].

Genetically, mature Natterin-1 and 2 are very similar, with 84% of identity to each other; and both orthologs have about 40% identity with Natterin-3 and 4. Natterin-P is the shortest among the founder members (71 amino acids in length) and shows a high homology (84% of identity), mainly with Natterin-4 in the first 55 amino acid residues within the *N*-terminal region [1].

Natterins-1–4 share a similar monomeric architecture, with two DM9 membrane-binding domains, which were primarily found in Diptera proteins with unknown functions, in the *N*-terminal region and an aerolysin domain in the *C*-terminal, similar to the common core of the aerolysin produced by *Aeromonas* species [4], which are essential for toxicity [2,3] and membrane penetration [5]. Notably, the Natterin members found in V*Tn* were classified as belonging to the aerolysin family of proteins [4].

The aerolysin-type family [4] form together with α-hemolysin family [6] and the MACPF/CDC family [7], the β-type pore-forming proteins (β-PFPs) superfamily, a useful set of molecular tools for molecular recognition, sensing, and cell signaling [8]. Aerolysin-like toxins have remarkable sequence identity or conserved structural elements similarity among their domains and are synthesized by a very diverse group of organisms throughout roughly all kingdoms of life. The pore-forming domain of aerolysin contains a flexible insertion loop, long thought to be the region that reorganizes into a β-hairpin during membrane insertion due to its amphipathic character of alternating hydrophilic/hydrophobic residues [4].

Typically, the module in *N*-terminal domains of aerolysin-like proteins have been demonstrated to provide binding specificity to target membrane receptors, triggering oligomerization of the aerolysin module in the *C*-terminal domain, which inserts into the lipid bilayer to allow electrolyte leakage, disruption of membrane function [9,10,11] and induction from the cell death to cell survival [12], being able to activate the NLRP3 inflammasome-dependent caspase-1 maturation and leading to IL-1β-dependent neutrophilic inflammation [13].

Following the first identification, the group of Natterin-like proteins has been expanded over time. Several sequences homologous to Natterin have been identified in different teleostean genomes, such as the venomous catfish *Plotosus lineatus* [14] and non-venomous fish, including lampreys-*Lampetra japonica* or *Lethenteron camtschaticum* [15] and *Lampetra morii* [16], arctic charr *Salvelinus alpinus* [17], zebrafish *Danio rerio* [10], atlantic cod *Gadus morhua* [18], and ovate pompano *Trachinotus ovatus* [19].

It has been suggested that the duplication of all genes through whole-genome duplications (WGDs) have played an important role in species radiation and environmental adaptation, including marine environment inhabitants, by offering a particularly remarkable opportunity for the emergence of evolutionary novelties and functional diversification [20]. The occurrence of Natterin-like proteins in a wide range of fish as well as in divergent aquatic species such the acroporid coral *Acropora digitifera* [21], the zebra mussel *Dreissena polymorpha* [22], and in pacific oyster *Crassostrea gigas* [23,24] highlights that the offensive role as a toxin for prey capture and defense of the territory is one of several functions acquired in addition to the function as an effective molecule in immune defense, which has not been lost.

The evolutionary conservation of the core aerolysin motif throughout all kingdoms of life reflects the biological relevance of these proteins. It could be hypothesized that the fish Natterin-like proteins derive from an ancestral protein of living cells considering its wide spectrum of distribution. However, the differences among those proteins and experimental observations support that recurrent horizontal gene transfer (HGT) events of genes encoding pore-forming toxins have happened several times independently. Therefore, both processes might have occurred in parallel and played vital roles in this group of protein dissemination.

In this work, we performed a detailed screening using available genome databases across a wide range of species to identify sequence members of the group of Natterin-like proteins to create phylogenetic relationships with *T. nattereri*, multiple sequence alignment, and conserved domain analysis. This is the first study that describes the overall distribution of Natterin-like proteins and extends the information about the domain architectures, conserved motifs located in the aerolysin domain for pore formation, and consequently reveals and clarifies specific biological roles.

## 2. Results and Discussion

### 2.1. Phylogenetic Analysis of Natterin-like Proteins

The investigation for Natterin and Natterin-like proteins in databases generated 769 results by NCBI from 278 species (96 fish). Ensembl presented 167 gene records, all identical with NCBI, which is a cross base. In the Uniprot, after discarding the duplicates, there were 90 Natterin-like proteins from 47 species (10 fish). From the literature, six species were added to the dataset, and three were fish (only information about the presence, not sequences). Overall, the data survey came up with 331 species, including Natterin founder members and 859 putative Natterin-like genes. The higher number of genes in relation to the species is because one organism generally presents more than one protein-coding gene (Table S1). Our interest was a survey about the diversity, and specific exploration, such as structural and conserved domain analysis, especially in fish (Table S2). Since Natterin proteins were first isolated in a fish, we decided to collect their protein sequences separately from the total diversity of species present in this group of proteins. It resulted in a list of 109 species (33% of total) with 598 protein sequences, with specimens from the primitive jawless fish group, to lobe-finned and ray-finned fish, the last that corresponded to the majority (Table S3).

A large number of proteins containing the Natterin domain are distributed throughout most of the kingdoms of life, except Prokaryotes, Protists, Amphibians, and Mammals, which corroborates what has been described for other sequences. Many types of species also contain a substantial number of proteins with few or no known metazoan homologs [25,26,27,28,29,30].

**Figure 1:**
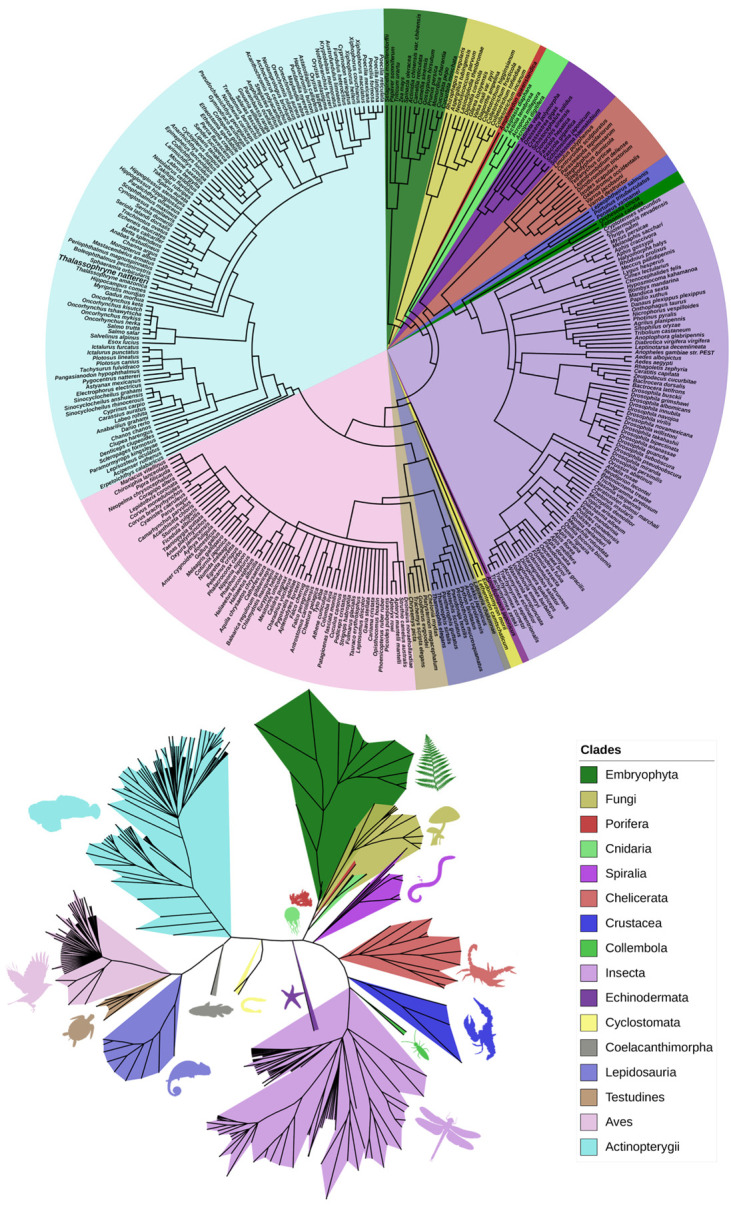
Phylogenetic trees generated using the software PhyloT that represent all the species found sharing any Natterin or Natterin-like protein in the tree of life. The circular tree with the corresponding species (**top**) and the unrooted tree with the main clades (**bottom**) demonstrate the species’ evolutionary relationship.

The phylogenetic analysis with all sequences yielded 16 major clades with considerable diversity of Eukaryotic organisms, including Plants, Fungi, and sessile marine animals with a primitive structure and anatomical organization, in addition to a large variety of fish that might be the animals presenting higher complexity within this group of proteins. The groups with more species included in our analysis were Insects, Birds, and Fish (Figure 1), in contrast to the majority (90%) of the aerolysin proteins found in Proteobacteria, Firmicutes, and Fungi [4].

The group of Natterin-like proteins might have somehow appeared in a distant ancestor of the Plants and Animals at least 400 million years ago. This is assumed once it is the most reasonable estimation of the first lineages’ divergence that presented any putative Natterin protein in our research. In addition, horizontal transfer might have helped to spread the proteins and increase their variability. Given its appearance in such a variety of taxons, as plants or venomous animals, we can attribute to the members of the Natterin group a decisive evolutionary role.

Based on the species from different taxons that present Natterin proteins, phylogenetic trees, also known as cladograms, were generated to represent evolutionary relationships among organisms based on clade grouping and their paths throughout the evolutionary process [31]. The most accurate phylogenetic tree will have the fewest nodes. This is called parsimony, which means that the best tree is the simplest [32].

**Figure 2:**
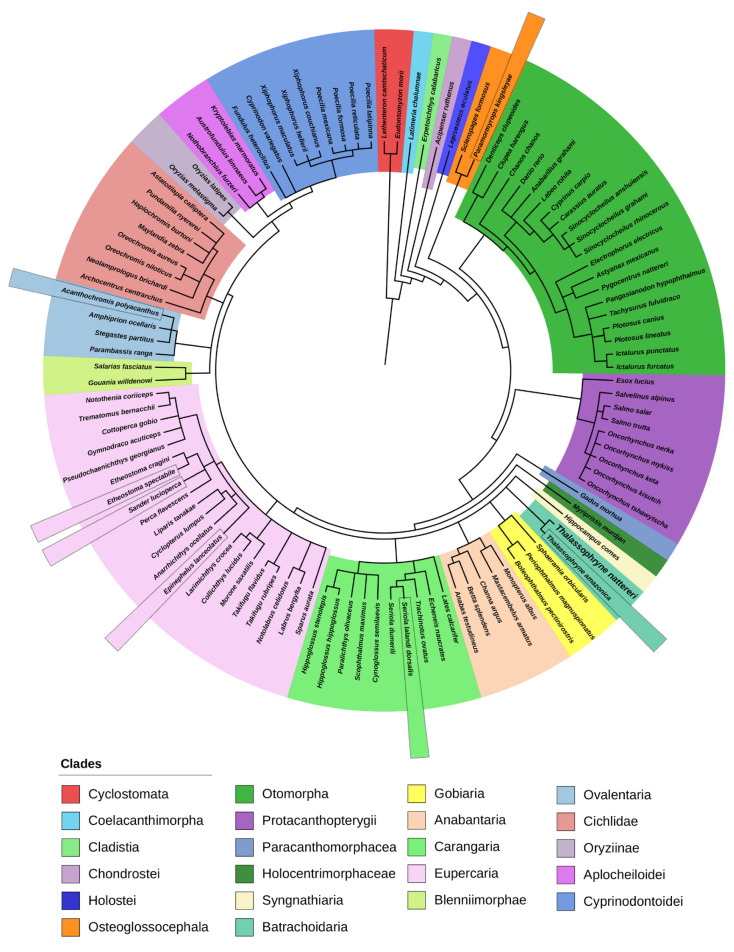
Phylogenetic tree generated using the software PhyloT to represent exclusively the fish species included in the group of Natterin-like proteins, 109 representatives. The species that presented the protein sequences with the higher identity percentage with the founder members Natterin-1–4 are highlighted.

It is essential to understand that phylogenetic trees are nested hierarchies, i.e., any individual set of branches is also part of a larger set of branches. This is easily seen in Figure 1 and Figure 2 and Figure S1, where, for example, *Thalassophryne amazonica* and *T. nattereri* are grouped in the clade of the Batrachoididae family. However, they are part of the Teleostei and Chordata clades as well. The Darwinian hypothesis of descent with modification predicts that a set of nested hierarchies would represent the organisms’ evolutionary history. 

In the general phylogenetic tree (Figure 1), it is notable that the Embryophyta and Fungi are in the tree base. Together, with all the other metazoan species, they make the representatives of the group of Natterin-like proteins. The relatedness comparison might be evidence that Natterin proteins arose long in the past, possibly in a pluricellular eukaryote. That inference came out because the most primitive species in the tree is *Selaginella moellendorffii*, which belongs to the Lycopodiophyta clade, considered the oldest division of existing vascular plants, including some of the most basal living species known that first appeared in the fossil record around 400 million years ago. The lycophyte *S. moellendorffii* is an important model organism in comparative genomics [33].

The Natterin domain was reported in ancestral species that belong to the older diverged lineages in Metazoans, e.g., the sponge *Amphimedon queenslandica*; an obligate biotrophic arbuscular mycorrhizal fungus *Rhizophagus irregularis*; the common Indo-Pacific scleractinian coral *Acropora digitifera*; the invasive bivalve species zebra mussel *Dreissena polymorpha*; common liver fluke *Fasciola hepatica*, blood flukes *Schistosoma japonicum* and *S. haematobium*; the blacklegged tick *Ixodes scapularis*; the salmon louse *Lepeophtheirus salmonis*; and the springtail *Orchesella cincta*.

Moreover, the tremendous contribution of the insects in the group of Natterin-like proteins was noticeable. Even though they are not closely related to fish, insects are the most diverse animals on the planet [34]. This would be enough to justify their overrepresentation in the search. As the largest and most widely distributed group of arthropod animals, invertebrates represent more than 70% of all species of living beings described. Insects were among the first animals to colonize and exploit terrestrial and freshwater ecosystems. These characteristics are undoubtedly related to their diversification [35].

Insects can be found in almost every ecosystem on the planet, and the most diverse orders are Odonata, Orthoptera, Lepidoptera, Diptera, Hemiptera, Coleoptera, and Hymenoptera. Our analysis showed the presence of Natterin-like proteins in four species of the *Apis* genus and in 16 species of *Drosophila*, except for *D. melanogaster*.

All the ray-finned fish share common ancestors with other groups like Lepidosauria, Testudines, and Aves. Although there were not many reptile species (14) in the whole group, the birds were very well represented, which might be explained by this evolutionary relationship. In addition, the birds form a diverse vertebrate group found all over the globe, from equatorial to polar regions. According to a study, the birds’ biodiversity is severely underestimated, and the authors determined that there are around 18,000 species worldwide, nearly twice as many as previously thought [36]. 

In the present study, we explored the fish clade since they are the main focus of our studies and experimental models. Indeed, in the aquatic species phylogeny, 86.5% of the organisms represented are fish (Figure 2). They represent more than half of the world’s known vertebrate species. Fish heterogeneity is based on many aspects of their biology and habitats. These differences evolved in parallel with the fact that fish have undergone a second WGD event (2R), following the ancient genome duplication that occurred in early vertebrates (1R) and a further one in the teleostean lineage (3R), all of these leading to the subsequent duplication or deletion of various genome parts [20,37,38,39].

The general classification of fishes is considered a paraphyletic assemblage, including the classes Myxini (hagfishes) and Petromyzontida (lampreys), from the superclass Agnatha; Chondrichthyes (sharks, rays, and chimeras); Sarcopterygii (coelacanths and lungfishes), and Actinopterygii (ray-finned fishes), from the superclass Osteichthyes (bony fish) (Figure S2) [37,40].

Other species from aquatic environments but fish are members of the phylum Porifera, Cnidaria, Protostomia, and Echinodermata (Figure S1). The Porifera representative is *A. queenslandica*, a sponge native to the Great Barrier Reef, the world’s largest coral reef system. Its genome was the first from a sponge to be sequenced, and it provides insights into the evolution of animal complexity and evolution of metazoan development [41].

Cnidaria is an Animalia phylum containing over 11,000 species; they are more complex than sponges and are found predominantly in marine environments. They mostly have two basic body forms: swimming medusae and sessile polyps, both radially symmetrical with mouths surrounded by tentacles that bear cnidocytes [42]. In our survey, four species of polyps were registered to contain Natterin-like. Although animal venoms have evolved at least a hundred times independently [43], evidence for the implication of horizontal gene transfer [44], including from parasitic fungi in the evolutionary origin of Natterin-like in the coral *A. digitifera*, has been issued by Gacesa et al. [45]. This type of mechanism that provides a quick channel for the evolution of novelty through the exploitation of bacterial or fungal weapons in animal venoms has also been shown to be crucial to centipede (Chilopoda) venoms [46], one of the oldest terrestrial venomous lineages, with a fossil record going back 418 million years.

The other aquatic non-fish clade composed of seven species in the Natterin group is the Protostomia, comprising animals with bilateral symmetry and three germ layers [47]. This group includes animals such as arthropods, annelids, and mollusks. Among the seven species shown here, there are rotifer, mussel, oyster, chelicerate arthropod, copepod, shrimp, and crab, indicating a great variety in the type of organisms. The Natterin domain was also found in one species of the Echinodermata phylum, the sea cucumber *Apostichopus japonicus* (Figure S1).

Interestingly, in the aquatic cladogram (Figure S1), the Cyclostomata and Coelacanthimorpha groups, considered the most primitive kind of living fish on Earth, share a common ancestor, not just with the modern ray-finned fish (Actinopterygii), but also with the Lepidosauria (one of the most prominent Reptilia groups, represented mostly by lizards and snakes), Testudines (an order of some of the earliest reptile alive, commonly known as turtles, tortoises, and terrapins), and the diverse class of Aves. These groups did not descend from each other but share ancestors and diverged through life’s evolution instead.

The Cyclostomata is a group of agnathans that comprise the living jawless fishes, with horny epidermal structures that function as teeth and branchial arches that are internally positioned instead of externally, as in jawed fish [39]. In the fish tree (Figure 2), Natterin-like sequences were found in Cyclostomates represented by the Arctic lamprey, also known as the Japanese river lamprey (*Lethenteron camtschaticum*, synonym *Lampetra japonica*) and the Korean lamprey (*Eudontomyzon morii*, synonym *Lampetra morii*), from the order Petromyzontiformes. These species represent the oldest fish to present Natterin-like proteins in this review. Most lampreys are ectoparasites on fish, using a circular, sucker-like mouth to clamp onto their hosts [48]. Unlike bony fish, lampreys lack scales, fins, and gill covers, however, like sharks, their skeletons are made of cartilage.

The lamprey clade likely diverged from a common ancestor in the Silurian Period, from 443 million to 416 million years ago [48]. It also corroborates with a time estimation of the raising of the Natterins since the Lycophytes and the Coelacanth divergence, according to genetic analysis of current species, are thought to have occurred about 390–420 million years ago [49,50,51].

Our search resulted in only one Sarcopterygii lineage species presenting a Natterin-like protein, the West Indian Ocean coelacanth *Latimeria chalumnae* considered phylogenetically closer to lungfish and tetrapods than ray-finned fish (Actinopterygii) [52]. The group’s most important characteristic is paired fins (pectorals and pelvic), whose bases are muscular peduncles that resemble the members of terrestrial vertebrates and move in the same way.

Together, Sarcopterygii and Actinopterygii form the group of bone-fish (Osteichthyes), which are more related to each other than to the lamprey group, and share a more distant ancestor and present very distinctive physical attributes. Natterin domain sequences were identified in only one species of the groups Cladistia, Chondrostei, Holostei, Paracanthomorphacea, Holocentrimorphacea, and Syngnathiaria, which are ramifications of the Actinopterygii (Figure 2).

However, it is crucial to notice that the fish in the bottom of the ray-finned clade are less related to most current fish since they diverged from the common ancestor long ago. The reedfish *Erpetoichthys calabaricus*, which lacks pelvic fins, is a member of the clade Cladistia; it consists of a few anguilliform (i.e., eel-shaped) remnants of an ancient diversity. The sterlet *Acipenser ruthenus* is the only member of the Chondrostei group to present Natterin-like genes. This is a group of essentially cartilaginous fish presenting some degree of ossification. Its members share with the Elasmobranchii (sharks and rays) certain features, such as the possession of spiracles, a heterocercal tail, and the absence of scales.

Holostei, an infraclass of the Neopterygii subclass, is represented in our cladogram by the presence of a Natterin-like sequence in the spotted gar *Lepisosteus oculatus*, restricted to the freshwaters of eastern North America [53]. Holosteans are closer to Teleosts than are the Chondrosteans. The spiracles are reduced to vestigial remnants (in gars, the spiracles do not even open to the outside), and the bones are lightly ossified. The thick ganoid scales of the gars are more primitive than those of the bowfin. A thin layer of bone covers a mostly cartilaginous skeleton in the bowfins, and they have many-rayed dorsal fins. In gars, the tail is still heterocercal but less so than in the Chondrosteans. 

The Teleostei is the most diverse lineage of the Neopterygii and by far the largest infraclass in the class Actinopterygii, from the 109 fish in the group of Natterin-like proteins, 106 are part of this group, as seen from the clade Osteoglossocephala (Figure 2). Teleosts are the most abundant aquatic vertebrates living today, containing over 30,000 named species [40], which is more than all living mammals, birds, reptiles, and amphibians combined. They comprise around 96% of all extant fishes and nearly half of all vertebrate species, which perhaps represents the most extensive adaptive radiation in vertebrate evolution [53,54]. The difference between Teleosts and other bony fish lies notably in their jawbones; they have a movable premaxilla and corresponding modifications in the jaw musculature, making it possible for them to protrude their jaws outwards from the mouth. Another difference is that the caudal tail fin’s upper and lower lobes are about equal in size. The spine ends at the caudal peduncle, distinguishing this group from other fish in which the spine extends into the upper lobe of the tail fin [55].

Resolution of the phylogenetic relationships of Teleosts is critical to understanding the timing of their diversification. There is currently a discordance between the estimated age of divergence for Teleosts, as inferred from the fossil record and molecular studies. It is estimated that crown teleosts’ lineage first diverged during the Carboniferous to early Permian (333.0–285.8 Ma), following the Devonian Age of Fishes [56].

More recent works connect the duplicate genomes of Teleosts as the driver of their prolific phenotypic diversification, concordant with the more general hypothesis that increased morphological complexity and innovation is an expected consequence of WGDs. This process provided entire sets of genes with increased biological complexity and the origin of evolutionary novelties [20,53,57]. The Teleost-specific (TS) WGD event, the third round in fish’s evolution, took place in the common ancestor of all extant Teleosts shaping this group’s history (Figure S2). The Teleost lineage split from basal ray-finned fishes and started to diverge after a WGD event that happened around 320–350 mya [38].

After WGD, duplicate genes (ohnologs) may follow different fates. The most likely outcome is that one member becomes a pseudogene and disappears (non-functionalization) due to the lack of selective constraint on preserving both, or the copies persist as a result of complementation [38,58]. Mechanisms that act on the preservation of duplicates are (1) subfunctionalization, which is the partitioning of different functions subsets of an ancestral among ohnologs, providing an attractive explanation for why so many duplicated genes exist in eukaryotes, without requiring each duplication event to have conferred a selective advantage [38,59]; and (2) neofunctionalization, a process where one ohnolog mutates into a function that was not observed before duplication, leading to the retention of both copies [38,60,61]. The new function must be positively selected; if only one ohnolog evolves a new beneficial function, however, it must also lose an essential ancestral function that the complementing ohnolog maintains; otherwise, the second copy will disappear because it is no longer positively selected [58]. Regarding the Natterin-like proteins, the last phenomenon might explain the broader number and diversity of functionalities potentially designated, primarily exhibited by fish.

Teleost fishes are adapted to widely varied habitats from cold Arctic and Antarctic oceans to desert hot springs; from fast, rock-laden torrential mountain streams to the lightless depths of ocean trenches [62]. Regarding their wide morphological variation, including not only torpedo-shaped fish built for speed, Teleosts can also be flattened vertically or horizontally, be elongated cylinders or take specialized shapes as in anglerfish and seahorses. The last example present in the Natterin group is the tiger tail seahorse *Hippocampus comes* (Syngnathiaria).

Classic explanations for Teleost success include key innovations in feeding (e.g., protrusible jaws and pharyngeal jaws), reproduction, and the modes that they use to take up, transport, and deliver oxygen to the tissues, and features that enhance the capacitance of blood for O_2_ (βb): the Bohr and Root effects, RBC β-adrenergic sodium proton exchangers (RBC β-NHE), and the retia mirabilia (teleost vascular countercurrent exchangers) [53,54]. As a consequence of the genomic rearrangements during the TS-WGD event, some immune molecular families have expanded tremendously in some species, providing important functional effects against pathogens to which different species have been exposed. Proteins of the Natterin-like group may be evidence of this diversification of function. The conservation of the Natterin domain in different species may have been crucial for the evolution of species.

Teleosts have adopted a range of reproductive strategies. Most use external fertilization without any further parental involvement. A fair proportion of Teleosts are sequential hermaphrodites, starting life as females and transitioning to males at some stage, with a few species reversing this process. The green swordtail shown in this research (*Xiphophorus helleri*) tends to undergo sex reversal under certain environmental conditions. Another example of species containing Natterin-like proteins, the mangrove killifish (*Kryptolebias marmoratus*), is the only naturally occurring vertebrate known to be capable of self-fertilization; most populations consist primarily or exclusively of hermaphroditic individuals or males, and females do not seem to exist [63].

Another curious example of a Natterin group member is the Amazon molly (*Poecilia formosa*), an all-female species thought to have originated due to hybridization between two other species in the genus. It reproduces gynogenetically, meaning once the sperm has penetrated the egg membrane, it takes no further part in the embryo’s development. The reproduction is triggered by copulation and stimulation by sperm from the males of other species in the genus [64].

According to the biological aspects of fish and environmental distribution, we observed that most species containing Natterin-like proteins are present in freshwater (~29%); followed by freshwater and brackish (~23%); marine (~21%); marine, brackish, and freshwater (~15%); or marine and brackish environments (~12%). It can be seen that half of the species occupy more than one environment throughout the life cycle. This is due to the tolerance to physical–chemical variations that some fish exhibit, as well as the behavior of moving into brackish or freshwater to spawn, such as *Morone saxatilis*. Still, some occupy different habitats at different life stages, such as *Hippoglossus stenolepis*, which when young are found near the shore, moving out to deeper waters as they grow older; besides this, no other freshwater fish are found as far north as the arctic charr *S. alpinus*, and among marine fish, it is notably a recurrent reef-associated behavior.

Within these aquatic environments, the species members of the Natterin-like group predominantly occupy the benthopelagic and demersal zones (~70%). Both are ecological regions associated with the lowest water body level, where the species live and feed near the bottom. The fish distribution through the climate zones was, in descending order, Tropical (~40%), Subtropical (32%), Temperate (~21%), and Polar (~6%), demonstrating the circumglobal distribution [65].

Regarding the potential to provoke envenomation in human victims, only four species are venomous and present venom apparatus: *Plotosus canius*, *Plotosus lineatus*, *Thalassophryne nattereri*, and *Thalassophryne amazonica*. Venoms, by definition, require a method by which their bearer can introduce them into the body of a target organism; this is accomplished via spiny elements associated with the fins or opercular and cleithral bones that contain grooves that facilitate the flow of venom along with the spin; in most cases, the glandular tissue rests within the groove itself [66]. 

The venom glands of catfishes (Plotosidae) are composed of aggregations of glandular cells associated with bony spines in the dorsal and pectoral fins. The spines of many species are additionally armed with retrorse serrations along one or both of the spine margins surrounded by a tegumentary sheath with specialized glands. When the spine enters a potential predator, the glands are torn, releasing the largely proteinaceous venom into the wound [66]. However, members of the genus *Thalassophyne* have a complete venom inoculation apparatus composed of two dorsal canaliculated spines and one on each side covered by a membrane and all connected to the venom glands at the base of their fins [67,68,69]. Two other species are poisonous when eaten; *Takifugu rubripes* and *Takifugu flavidus* contain lethal amounts of the poison tetrodotoxin in the internal organs, especially the liver and ovaries. Moreover, *Myripristis murdjan* and *Seriola dumerili* have been reported to provoke ciguatera poisoning, a foodborne illness.

Natterin-like sequences are observed in the three different species that can cause traumatogenic injury through bites *Pygocentrus nattereri*, *Epinephelus lanceolatus*, and *Anarrhichthys ocellatus* (Figure 2). Lastly, the electric eel, *Electrophorus electricus*, a South American electric fish and the only species in its genus, presents voltage electric organs that can discharge electric shocks [65].

Even though Natterin-like genes are widely distributed among different organisms, they do not appear homogeneously throughout evolution; mammals, for example, were not included in our results. Furthermore, bacteria did not show up in our search, despite the fact that the Aerolysin is the founding member of a major class of pore-forming toxins. Even the Chondrichthyes did not present Natterin-like sequences up to date, leading to the understanding that the Natterin-like proteins follow a distribution pattern where some groups and many species that descend from the same common ancestor do not have this type of gene.

Whether or not fish have evolved independently, the question of central importance is whether they preferentially retained Natterin-like as one more common solution to challenges of infections despite their exploitation of widely divergent trophic ecologies, consistent with continuity of function and adaptive value.

### 2.2. Multiple Alignments of the 15 Most Similar Members of Natterin-like Proteins

Next, we generated a multiple sequence alignment of Natterin-like sequences from the predicted aerolysin conserved domain limited to the inner β-barrel and the outer β-barrel region of the pore structure (residues 190–315 amino acids) of Natterin founder members to evaluate the conservation of protein domains, as well as individual amino acids or nucleotides [70,71].

**Figure 3:**
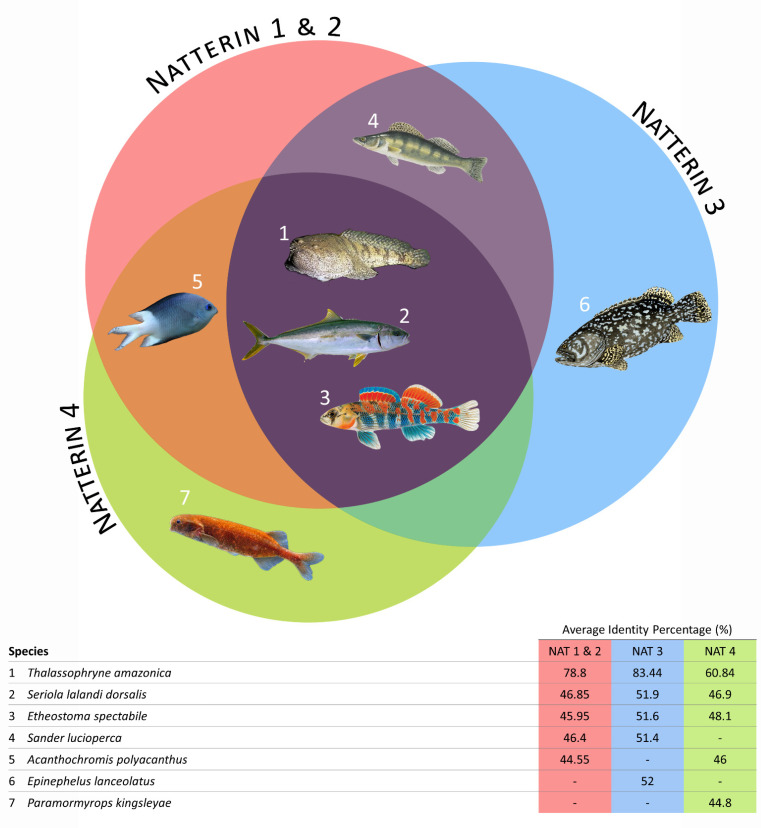
The seven fish species presenting the Natterin proteins (the topmost 15 sequences) with a higher percentage of identity (pid) compared with founder members Natterins-1–4 (**top**); The queried sequences’ average identity percentage comparing to the founder members Natterins-1–4 (**bottom**).

Initially, the alignment of all the Natterin-like protein sequences from the group of fish clustered together generated a wide range of sequence identity from 12% to 87% between domains. When we narrow the search for the top 15 protein sequences with the high level of identity with the Natterins from *T. nattereri*, we verified that except the *Sander lucioperca* (Natterin-1, Natterin-2, and Natterin-3) *Acanthochromis polyacanthus* (Natterin-1, Natterin-2, and Natterin-4), *Epinephelus lanceolatus* (Natterin-3), and *Paramormyrops kingsleyae* (Natterin-4), the species *Thalassophryne amazonica*, *Seriola lalandi dorsalis*, and *Etheostoma spectabile* showed sequences with a high identity with the all Natterin founder members (Figure 3). As expected, *T. amazonica* presented sequences with the highest percentages of identity, and all 15 sequences analyzed showed a greater identity with Natterin-3.

*Thalassophryne* is a genus of venomous toadfish found in the western Atlantic Ocean, and *T. amazonica* is found in the Amazon River and some of its tributaries, while *T. nattereri* has been found in Northeastern Brazil. *T. amazonica* presents two putative Natterin genes and 24 Natterin-like genes, four of them with two isoforms, totalizing 30 proteins. This finding is surprising because both descend from a common ancestor (Figure 2).

In Table S3, we observed 64 species with two to five genes, 25 species with six to nine genes, nine species with more than ten genes, and two species had more than 20 genes, suggesting evolutionary-driven gene duplication. Specifically, *T. amazonica* (26); *Anabas testudineus* (25); *Sinocyclocheilus grahami* (14); *Salmo salar* (13); *Oreochromis niloticus* (12); *Sinocyclocheilus rhinocerous* (12); *Notolabrus celidotus* (11); *Oncorhynchus mykiss* (11); *Perca flavescens* (11); *Acanthochromis polyacanthus* (10); and *Danio rerio* (10) are the species with the highest numbers of genes. In contrast, eight species presented only one Natterin-like gene, as follows: *Boleophthalmus pectinirostris*, *Latimeria chalumnae*, *Paramormyrops kingsleyae*, *Ictalurus furcatus (Pimelodus furcatus)*, *Lethenteron camtschaticum* (*Lampetra japonica*), *Eudontomyzon morii* (*Lampetra morii*), *Plotosus canius*, and *Trachinotus ovatus*.

This inconsistency in the number of copies is probably because it is estimated that 75% of the genes from the TS-WGD event may revert to singletons [72]. Contrarily, the duplication-degeneration-complementation (DDC) hypothesis [73,74] might help to explain the unexpectedly high retention of duplicate genes, which suggests that genes with simple tissue- and time-specific regulatory elements would be more likely to revert to singletons than those with complex regulation.

**Figure 4:**
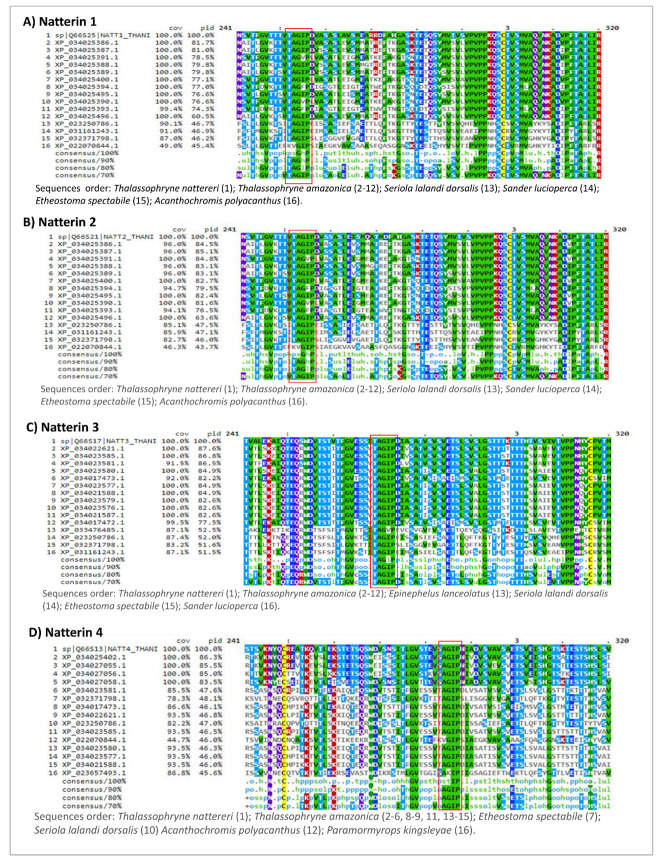
Multiple alignment and conserved residues found on the 15 topmost similar fish sequences compared with the founder members Natterins-1–4, based on the percentage of identity (pid). The conserved residues “TAGIP” and “AGIP” are highlighted in the pictures, picture (**A**–**D**), respectively.

To further characterize the Natterin domain, we explored separately Natterin-like sequences of *Thalassophryne amazonica* (XP_034025386.1; XP_034025387.1; XP_034025391.1; XP_034025388.1; XP_034025389.1; XP_034025400.1; XP_034025394.1; XP_034025495.1; XP_034025390.1; XP_034025393.1; XP_034025496.1; XP_034025402.1; XP_034027055.1; XP_034027056.1; XP_034027058.1; XP_034023581.1; XP_034017473.1; XP_034022621.1; XP_034023585.1; XP_034023580.1; XP_034023577.1; XP_034021588.1; XP_034023579.1; XP_034023576.1; XP_034021587.1; XP_034017472.1), *Seriola lalandi dorsalis* (XP_023250786.1), *Sander lucioperca* (XP_031161243.1), *Etheostoma spectabile* (XP_032371798.1), *Paramormyrops kingsleyae* (XP_023657493.1), *Epinephelus lanceolatus* (XP_033476485.1), and *Acanthochromis polyacanthus* (XP_022070844.1) to compare with Natterin-1 (Q66S25), Natterin-2 (Q66S21), Natterin-3 (Q66S17), and Natterin-4 (Q66S13) of *T. nattereri* (Figure 4).

Natterin-1 and Natterin-2 contain six evolutionarily conserved motifs in the interval (241–320 aa) GV, AGIP, QSY, VPVPP, MVA, and PFTATLIR. Natterin-3 shows eight evolutionarily conserved motifs QTEQRWDV, TST, GV, SS, AGIP, ETSLSVLGST, TTTHSV, and VTVPPN. In contrast, few motifs were found in Natterin-4, TK, VTL, WD, GV, AGIP, and ETS. Among all motifs, two motifs that remained present in all four Natterin sequences were GV and AGIP (Figure 4).

The AGIP motif is composed of four non-polar hydrophobic residues, which show metabolically inexpensive features. Alanine (A) and isoleucine (I) residues show a preference to be in regions inside the regular secondary structure. As expected, both glycine (G) and proline (P) also show a preference for regions outside the regular secondary structure and play important roles in many turn types. Proline (P) disrupts the secondary structure and is often found as a capping residue. Glycine (G) is often found in loop regions, probably because of a conformation with positive φ (*phi*, torsion angle around the N–C_α_ bond) that is often required to complete a turn, as reviewed by Shapovalov, Vucetic, and Dunbrack [75].

Natterins-1–4 show four glycine (G) residues along the length of the pore-forming region, including one of them within the AGIP motif and a second along with the non-polar hydrophobic valine (V) residue forming the short GV motif (Figure 4), described as the main residues that act as hinge located on the membrane-binding domain involved in the pre-pore to pore conformation [76]. GV and AGIP motifs remained preserved in all top 15 Natterin-like proteins as in most of them. Then, we reasoned that both motifs represent to members of the group of Natterin-like proteins the pore-forming loop for membrane anchoring ability and transmembrane barrel insertion.

A special feature of all β-PFPs is alternating serine (S) and threonine (T) polar residues found in the insertion loop, as well as throughout the rest of the pore-forming modules, such as the lumen of the β-barrel. These residues are thought to participate in membrane binding [77] and oligomerization [78], and help the amphipathic loops in transmembrane pore formation [79].

Interestingly, the sequence consensus AGIP present in Natterins-1–3 founder members is immediately flanked by threonine (T) and aspartate (D) residues on each side and beyond to the end of the *C*-terminal by one residue of serine (S), and farther away from this site surrounding by other flexibility-inducing amino acids (serine or threonine), characterizing a region rich in carboxyl (aspartate) and hydroxyl (serine or threonine) groups allowing interactions that guarantee the flexibility and stabilization of the loop conformation. However, in Natterin-4, the threonine (T) and aspartate (D) flexible residues surrounding the AGIP core have been replaced by serine (S) and polar asparagine (N), and the presence of threonine (T), aspartate (D), and serine (S) residues were noticed to be flanking this structure on each side. Asparagine (N) and aspartate (D) are known to adopt conformations in the left-handed α-helical region and other partially allowed regions of the Ramachandran plot more readily than any other non-glycyl amino acids [80].

Then, we found that the two hydroxylated amino acid residues of threonine (T) and serine (S) that flanked the AGIP motif located in the insertion membrane of Natterin-1 and Natterin-2 was conserved in all sequences, except in Natterin-like sequences of *Acanthochromis polyacanthus* that was replaced by lysine (K) and glutamic acid (E) residues, which demonstrated low and intermediate flexibility features, respectively [81]. In comparison to Natterin-4, the threonine (T) residues were replaced by serine (S) in seven Natterin-like sequences of *T. amazonica* and one sequence of *Paramormyrops kingsleyae*. The serine (S) residues were replaced by lysine (K), glycine (G), and mainly threonine (T) in sequences of *T. amazonica* (7 sequences)*, Etheostoma spectabile* (1 sequence), *Acanthochromis polyacanthus* (1 sequence), and *Paramormyrops kingsleyae* (1 sequence). However, all Natterin-like sequences conserved both polar residues when compared to Natterin-3.

The aspartate (D) residue surrounding AGIP present in Natterins-1–2 involved in intramolecular hydrogen bonds was replaced by a flexible leucine (L) residue in three sequences of *T. amazonica*, by glutamic acid (E) in one sequence of *Seriola lalandi dorsalis* and *Sander lucioperca*, and by serine (S) in one sequence of *Etheostoma spectabile* and *Acanthochromis polyacanthus*. Compared with Natterin-3, this residue was replaced by glutamic acid (E) in *Epinephelus lanceolatus, Seriola lalandi dorsalis*, *Sander lucioperca*, but in *Etheostoma spectabile* it was replaced by serine (S). In addition, the asparagine (N) residue in Natterin-4 was replaced in seven sequences of *T. amazonica* by aspartate (D), in one sequence each of *Etheostoma spectabile* and *Acanthochromis polyacanthus* by serine (S), in one sequence of *Seriola lalandi dorsalis* by glutamic acid (E), and in one sequence of *Paramormyrops kingsleyae* by the isoleucine (I) residue (Figure 4).

Mayorov, Dal Peraro and Abriata [81] demonstrated that sites that favor flexibility display variable degrees of solvent exposure and intermediate to high conservation, suggesting their relevance for protein fitness. Flexibility might be required to modulate receptor selectivity, and binding stabilized by hydrophobic contacts and rigid amino acids. In this view, we suggest that the substitutions which occurred in these sites (Natterin-1: GV**T*TAGIPD**S*S, Natterin-2: GV**T*TAGIPD**S*S, Natterin-3: GV**S*TAGIPD**S*T, and Natterin-4: GV*T**SAGIPN**D*S) have no potential to evolve new functions and traits, since the mutations were not deleterious.

Individual amino acid residue properties (e.g., small or large volumes, metabolic cost, hydrophobicity or flexibility capacity, among others) can reflect functional roles. In general, the occurrence and the relative positions of cysteine residues are recognized as important factors in both protein structure and function. This is because of their ability to form intra- and inter-molecular disulfide bridges that influence protein folding, thus affecting protein’s functionality. We analyzed in this interval (241–320 aa) the presence of the only conserved cysteine (C) at the distal tip of the pre-forming domains of Natterins-1–4 and observed that all 15 sequences conserved the cysteine residue, consistent with a role in loop stabilization.

Together, the details of amino acid-conserved residues and non-deleterious punctual diversity that were deduced among species have outlined molecular determinants. The magnitude of change likely reflects molecular elements that have figured decisively in both the lineage and species evolution of the Natterin proteins.

Although bacterial aerolysin-like toxin structures were characterized in their soluble form [77,78,82,83,84], revealing a mushroom-like structure with a central stem built of a β-barrel from the top to the bottom of the pore, the only Natterin-like protein structure that had the pore-forming mechanism unveiled was the dimeric protein encoded by *Aep-1* (previously termed *Dln1*) from *Danio rerio*. Using X-ray crystallography and low-resolution cryo-electron microscopy structure, Jia et al. [10] described that the vertebrate Natterin-like *Aep-1*, in a different way from bacterial aerolysin, forms ring-shaped octameric pre-pores aligned in parallel with 38 Å of a diameter at the gate of the central hollow and 26 Å at the bottom. The oligomerization of Natterin-like *Aep-1* is triggered after binding to the *N*-terminal to Manα1-2Man and Manα1-3Man-binding site receptors of yeast cells in a pH-dependent manner. The aerolysin module of *Aep-1* bends toward the lectin module in contrast to the *A. hydrophila* aerolysin module, which flattens with respect to the receptor-binding domain to a position almost parallel to the membrane during the pre-pore to pore transition, accompanied by a vertical collapse of the heptamer [79].

**Figure 5:**
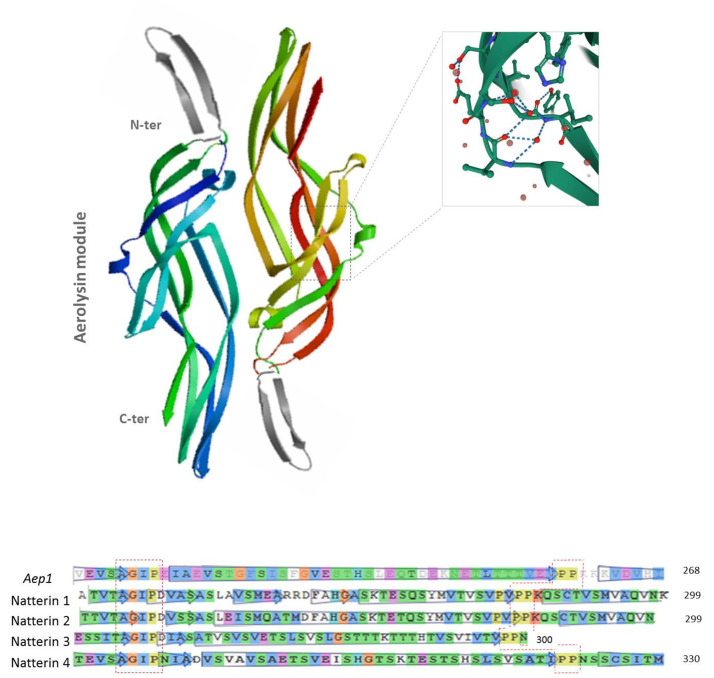
Model built by SWISS-MODEL representing the overall structure of founder Natterins-1–4 from *Thalassophryne nattereri*. In the top, cartoon representation of Natterin proteins using as template the crystal structure of *Dln1* dimer (PDB 5DI0), a Natterin-like protein of *Danio rerio* (Method: X-Ray diffraction; resolution: 1.70 Å; R-value free: 0.209; R-value work: 0.172; R-value observed: 0.174; deposited by Jia et al. [10]). The Aerolysin module shared by all founder proteins is colored whereas the additional modeled *N*-terminal portion present only in the founder Natterin-1 and -2 is presented overlapped in gray. The zoomed part within the 3D representation shows the AGIP motif localization, which remains preserved in all Natterin-like proteins. In the bottom, multiple-sequence alignment of an Aerolysin module segment highlighting the conserved motifs shared by the template and the four founder members: AGIP and PP.

Next, we performed the protein structure prediction and homology modeling of Natterins (Figure 5). Initially, the four founder members of the group of Natterin proteins (Accession-UniProt Q66S25.1 for Natterin-1, Q66S21.1 for Natterin-2, Q66S17.1 for Natterin-3, Q66S13.1 for Natterin-4) were modeled using Aerolysin chain A from *Aeromonas hydrophila* (UniProt P09167) performed by SWISS-MODEL. Natterin-1 and -2 had the same shape as the template but were different from Natterin-3 and -4, which were similar to each other (data not shown).

Later, they were modeled using Natterin-like *Aep-1* dimer (PDB-5DIO) structure [10] from zebrafish, which has a greater evolutionary relationship with the *T. nattereri.* Notably, *Aep-1* and Natterins-1–4 do not vary significantly in the insertion loop sequences, similarly rich in serine and threonine residues, most of which are found flanking the highly conserved AGIP motif (Figure 5).

Due to the homology shared between Natterin-like *Aep-1* and the founder members of the Natterin group, we expect that the other Natterin-members who have the conserved motif AGIP follow similar strategies for the formation of the β pore barrel. However, it remains to be characterized the pore-forming mechanisms within the group of Natterin-like proteins and whether the members have evolved variations of the common conformational strategy and how these relate to their respective physiological role, including immunity, signaling, and development.

### 2.3. Conserved Domains of Natterin-like Proteins

All Natterin-like proteins share a similar architecture with a membrane-binding domain and a structurally conserved pore-forming region. The group of Natterin-like proteins exhibit variable *N*-terminal modules and a remarkably high degree of similarity in their pore-forming domains (Tables S2 and S3).

**Figure 6:**
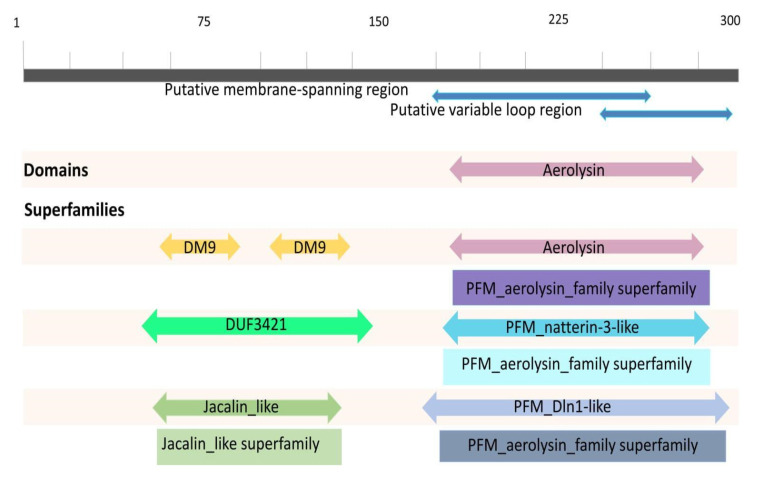
The founder members Natterins-1–4 conserved domains followed by the most found conserved domains and their superfamilies in the fish protein sequences in the group of Natterin-like proteins using the NCBI Conserved Domains Tool.

Sequence analyses revealed different domain rearrangements among the fish Natterin-like sequences. Based on all the information retrieved, four main types of architecture can be distinguished (Figure 6). The combination of the aerolysin domain linked to DM9 was described for the founder members of the group (Natterins-1–4) [1]. Of the total 598 sequences in fish, 44 presented only the aerolysin domain and 69 sequences showed a combination of the aerolysin domain linked with the jacalin-like domain. The majority (477) of sequences presented a combination of the aerolysin domain linked to the DUF3421 domain. However, a few sequences presented the aerolysin domain linked to several others (e.g., Nucleotidyl Transferase domain, Caspase activation and recruitment domain, and Death Domain).

DUF3421, classified among the proteins of an unknown function, is a family of proteins harboring repeats of the DM9 domain, a 60–75 amino acids motif first described in a small number of *Drosophila melanogaster* proteins [85]. DUF3421 proteins may carry two or more DM9 domains either in combination with other domains or as their sole constituent, like in the *Anopheles* genus that has four DM9 repeats [86].

Recently, a protein harboring two DM9 repeats was identified as mannose-specific lectin (CgCGL1, renamed as CgDM9CP-1) from the hemolymph of the Pacific oyster *Crassostrea gigas* [23,24]. Later, Liu et al. [87,88] provided further evidence for the function of the DM9 domain in the innate immune system. They found in *C. gigas* another two DM9s containing proteins designated as CgDM9CP-2 and CgDM9CP-3, acting as a pattern recognition receptor with a broad range of recognition spectrum.

*C*-type carbohydrate recognition domains (*C*-type CRDs) have been characterized into two groups according to their motif, which can be mannose (Man)-binding EPN (Glu-Pro-Asn) motif or galactose (Gal)-binding QPD (Gln-Pro-Asp) motif [89]. However, variations of these motifs, resulting from a mutation of a single amino acid, were reported; in shrimp, *C*-type lectin (CTLs) variants include EPD, EPK, EPQ, EPS, QPE, QPG, QPN, QPS, QPT, QYE, YPD, YPG, and YPT [90]. Recently, another binding motif variant with a QAP (Gln-Ala-Pro) sequence specific to Gal has been reported in several shrimp CTLs [91]. 

Interestingly, our sequence analyses revealed that none of the mannose or galactose binding motifs are present in DM9 domains of Natterins-1–4. Moreover, no putative integrin-binding motif was located at the *N*-terminus of these proteins, the canonical sequence RGD (Arg-Gly-Asp), KGD (Lys-Gly-Asp), or YGD (Tyr-Gly-Asp).

The β-prism-I lectins, which are also commonly known as the jacalin-related lectin (JRLs) family, derive their name from jacalin, the first member to be identified from the seeds of jackfruit, *Artocarpus integrifolia* [92]. The Jacalin domain consists of a threefold symmetric β-prism made of three four-stranded β-sheets that preferentially bind to complex glycans rather than to simple mono- and oligosaccharides. Jacalin-like proteins are involved in many more biological processes related to stress signal transduction and defense. They can fulfill specific functions inside the host or in the interaction with other organisms [93].

Indeed, genome-wide investigations performed here of amino acid sequences revealed that the group of Natterin-like proteins are more abundant than expected and are more widespread in the eukaryota taxons. The changes in the binding-receptors specificity of the *N*-terminal modules of these members could also ensure their interaction with different molecules resulting in other interactions given the diversity of environments and pathogens to which different species have been exposed. In addition, the Natterin module ensures cell signaling for an immune protective outcome.

### 2.4. Functional Analysis in the Immune Response

Bacterial aerolysin β-PFPs are destined to kill the cells of host organisms or to have roles in interspecies relations [94,95]. Eukaryotic members of aerolysin β-PFPs serve in defense against pathogens or parasites, such as enterolobin [96] or amaranthin-like proteins [97] from plants, lysenin from earthworm [98], or assist in prey digestion, such as hydralysins from green hydra [99].

In this context, although biological functions for Natterin-like proteins remain uncharacterized, several studies have demonstrated their involvement as effector molecules in the host immunity. Upregulation of Natterin-like proteins after bacterial or viral infections was observed in the blood, heart, liver, intestine, or lymphoid tissues (gills, head kidney, skin, and spleen) of atlantic salmon *Salmo salar* [100], common carp *Cyprinus carpio* [101], adult zebrafish [102], zebra mussel [103], and lamprey [16].

Natterin-like proteins have been identified as another component of the skin, an immune-related tissue, important in the innate immune response, especially during the early stages of development [104,105]. Cokus et al. [106] identified a differential expression of Natterin-like *Aep-1* according to each stage of embryonic development of zebrafish in the outer periderm and inner basal cell layers, which have distinct properties, functions, and fates. The *Aep-1* gene was highly expressed at 52 (when the two epithelial layers are established) and 72 h post-fertilization (hpf) (when both layers have matured) than 20 SS (20 somite stage occurs at approximately 19 hpf in embryos, when the two epithelial skin layers are not yet fully defined), which was not previously reported to be expressed in the skin during early development.

Moreover, Natterin-like proteins have been explored as drug-delivery system tools in biotechnological applications for cancer treatment [107,108].

Investigations with the Natterin founder members through in vitro approaches and in vivo studies have allowed our group to extend their knowledge regarding the diversity of their functions and have shown that apart from its toxic effects, Natterins act as protease-degrading extracellular matrix components (type I and type IV collagens) promoting necrosis and cell detachment [109].

In the context of the assignments of proteinase activity, many environmental allergens from diverse sources have this activity, which has been suggested to skew the immune response toward the Th2 phenotype. We demonstrated that the proteolytic activity of Natterins, besides inducing a Th2 response with plasmatic titers of high-affinity antigen-specific IgEs over extended periods, is sufficient for the generation of survival signals that contribute to the formation of a molecular survival niche in the spleen and is essential for the longevity of the long-lived antibody-secreting cells with the B220^neg^ phenotype [110].

Later, Komegae et al. [111] highlighted the involvement of TLRs in controlling the overall magnitude of memory response induced by Natterins, especially the relationship between B cell migration and differentiation and the persistence of a distinct subtype of B cells into specific tissue niches. We demonstrated that TLR4 regulates the degree of expansion of memory B cells in the peritoneum (MyD88-dependent) and bone marrow (MyD88-independent), as well as in long-lived antibody-secreting cells in the spleen (MyD88-independent). TLR2 regulated the intensity of the expansion of memory B cells (independent of MyD88) and intermediated antibody-secreting cells (MyD88-dependent) in the bone marrow.

NLRP3, the most extensively studied member of the inflammasome family, has been implicated in sensing a multiplicity of pathogens like bacteria, virus, fungus, parasites, and several aerolysin-like pore-forming toxins from bacteria or viruses [112,113]. In response to cellular stress, the NLRP3 inflammasome activates multimerization of the adaptor molecule ASC (apoptosis-associated speck-like protein with a caspase recruitment domain) and pro-caspase-1, resulting in the processing and secretion of the pro-inflammatory cytokine IL-1β [114].

More recently, we extended the data that showed NLRP3 as the only member of the inflammasome family implicated in the sensing of several aerolysin-like pore-forming toxins [113,115,116,117,118] and we emphasized that the NLRP6-dependent neutrophil-mediated response may be part of the innate immune mechanism of the antimicrobial response of fish.

We confirmed Natterin from the V*Tn* as a pro-inflammatory molecule inducing in mice local and systemic neutrophilic inflammation dependent on the signals derived from IL-33/ST2 and IL-1β/IL-1R1, as well as IL-1α. Interestingly, the Natterin-dependent neutrophilic inflammation was mediated by the activation of both caspase-1 and caspase-11 by the non-canonical NLRP6 and NLRC4 adaptors through ASC interaction of the inflammasome complex, independent of NLRP3 [119].

These data show the ability of Natterin proteins to interact with other classes of innate receptors and thus maximize the immune response against antigens and pathogens. Natterin proteins are potent pro-inflammatory molecules and the data presented here is evidence that a large number of cells may sense and respond to Natterin.

## 3. Final Considerations

The set of results presented here shows a large number of Natterin-like sequences in the domain Eukarya that originated at least 400 million years ago and pointed to the importance of the evolutionary conservation of the aerolysin module across the Natterin group. The broad diversity of this group of proteins, notably in fish, might be due to the neofunctionalization process the extra copies provided by genome duplication underwent. It also corroborates the wide variety of fundamental roles they express in different organisms well beyond toxins, mainly in a complex apparatus such as the immune system. The similar domain structure suggests a similar pore formation mechanism for members of the protein group and mainly highlights a common full-fledged new functional role preserved over time. We understand that the members of the group play a highly specific role in the maintenance of species up to today for providing them the ability to defend themselves against unfavorable circumstances such as abiotic stress, pathogens, and parasites present in different environments to which they are exposed. In conclusion, the proteins of the Natterin group can be considered crucial constituents of the innate immune defense system. They are expressed during different developmental stages and act as effector molecules, activating immune cells by interacting with conserved intracellular signaling mechanisms in the hosts.

## 4. Methods

### 4.1. Protein Diversity and Phylogenetic Analysis

We used some of the most relevant and complete genome browsers in terms of diversity of species and coverage to collect the Natterin and Natterin-like genes distributed and annotated throughout the tree of life and evolutionary history. The search term “natterin” was looked up in the National Center for Biotechnology Information (NCBI) platform (https://www.ncbi.nlm.nih.gov/, accessed on 17 September 2020) with overlapping checking in the Ensembl Genome Browser (http://www.ensembl.org/index.html, accessed on 17 September 2020). The findings were complemented with other Natterin-coding genes collected from UniProtKB-Protein knowledgebase (https://www.uniprot.org/, accessed on 17 September 2020) after rejecting duplicates. Additionally, we searched in the literature, through Pubmed (https://pubmed.ncbi.nlm.nih.gov/, accessed throughout October 2020), ScienceDirect (https://www.sciencedirect.com/, accessed throughout October 2020), and Google scholar (https://scholar.google.com.br/, accessed throughout October 2020) for published articles describing species with genes from the Natterin group and not listed in the databases previously accessed. A list of species presenting one or more genes in the group of Natterin-like proteins was generated, including information about the gene number, description, IDs, habitats, and lineage.

Furthermore, the set of organisms listed in the review were grouped into three categories: general, including all living beings, organisms restricted to aquatic habitats, and solely fish. The last two categories were refined since the organism in which this group of proteins was discovered is a fish. This separation was useful for generating three cladograms, to visualize the evolutionary relationship among species presenting these proteins, and proceeding with the structural and functional investigation. The phylogenetic trees were generated using the software PhyloT v2 (https://phylot.biobyte.de/, accessed on 8 December 2020), a phylogenetic tree generator based on NCBI or Genome Taxonomy Database (GTD). These trees were displayed by the Interactive Tree Of Life (iTOL) system (https://itol.embl.de/, accessed on 8 December 2020) [120].

### 4.2. Multiple Sequence Alignment

For the multiple alignments, we used 594 sequences corresponding to all the proteins and their isoforms exclusively from fish in the group of Natterin-like proteins compared with the complete sequences or C-terminal region of the Natterin proteins of Thalassophryne nattereri. The protein sequences were obtained from the genome browsers and aligned utilizing the multiple sequence alignment tool from the software Clustal Omega-European Molecular Biology Laboratory/The European Bioinformatics Institute (EMBL-EBI) (https://www.ebi.ac.uk/Tools/msa/clustalo/, accessed on 20 January 2021). Clustal Omega is a new multiple sequence alignment program that uses seeded guide trees and profile hidden Markov model (HMM) techniques to generate alignments among sequences. Profile HMMs turn a multiple sequence alignment into a position-specific scoring system, aligning sequences and search databases for remotely homologous sequences. The alignment was visualized in the viewer MView (https://www.ebi.ac.uk/Tools/msa/mview/, accessed on 20 January 2021). All variations of the Clustal software align sequences using a heuristic that progressively builds a multiple sequence alignment from a series of pairwise alignments. Essentially, Clustal creates multiple sequence alignments through three main steps: performing a pairwise alignment using the progressive alignment method; creating a guide tree; and using the guide tree to carry out multiple alignments.

### 4.3. Structural Analysis of Conserved Domains

The domains can be identified as blocks of structural motifs or sequences recurrent in studied proteins when performing bioinformatic analysis of protein sequences. The conserved domains within the fish protein sequences were explored using the NCBI tool Conserved Domains (https://www.ncbi.nlm.nih.gov/Structure/cdd/wrpsb.cgi, accessed on 8 February 2021), applying the protein primary sequences IDs [121,122,123,124]. The most significant hits that covered large parts of the sequence were used in the comparison and recorded.

## Data Availability

Not applicable.

## Supplementary Materials

The following are available online at The following are available online at https://www.mdpi.com/article/10.3390/toxins13080538/s1, along with copies of the figures embedded in the text in higher resolution. Table S1: List of the all species included in the group of Natterin-like proteins with their respective Natterin-like genes information, and biological features from databases of NCBI, Ensembl, and Uniprot. Table S2: List of the fish species included in the group of Natterin-like proteins with their respective Natterin-like genes, proteins/isoforms IDs and primary sequences, biological features, percentages of identity with founder members, and conserved domains. Table S3: List of the fish species included in the group of Natterin-like proteins with their respective Natterin-like genes and proteins/isoforms IDs. Figure S1: Phylogenetic tree generated using the software PhyloT to represent the aquatic species included in the group of Natterin-like proteins. There are representatives from Porifera to ray-finned fish (Actinopterygii). Figure S2: The general classification of fishes, a paraphyletic assemblage including the Agnatha, Chondrichthyes, Sarcopterygii, and Actinopterygii. Groups uncolored do not have representatives included in the group of Natterin-like proteins. The three rounds of whole-genome duplication (WGD) events are pointed to in the cladogram.
