# Supplementary material for: The Natterin Proteins Diversity: A Review on Phylogeny, Structure, and Immune Function
Source: Toxins (Basel). 2021 Jul 31;13(8):538. doi: 10.3390/toxins13080538 (PMC8402412; doi:10.3390/toxins13080538)
Supplement: Supplementary file 1 [file toxins-13-00538-s001.zip › toxins-1252858 Supplementary Materials.pdf]

Supplementary Materials: The Natterin Proteins Diversity: A Review on Phylogeny, Structure, and Immune Function

Carla Lima, Geonildo Rodrigo Disner, Maria Alice Pimentel Falcão, Ana Carolina Seni-Silva, Adolfo Luis Almeida Maleski, Milena Marcolino Souza, Mayara Cristina Reis Tonello and Monica Lopes-Ferreira

Table S3. List of the fish species included in the group of proteins Natterin with their respective Natterin-like genes and proteins/isoforms IDs.

Scientific name	Gene symbols	Protein and Isoforms ID
		**NCBI**
Acanthochromis polyacanthus	LOC110972886; LOC110971679; LOC110965958; LOC110965957; LOC110965951; LOC110965950; LOC110965949; LOC110965948; LOC110965947; LOC110945731	XP_022078957.1; XP_022077763.1; XP_022070850.1; XP_022070849.1; XP_022070845.1; XP_022070844.1; XP_022070843.1; XP_022070842.1; XP_022070841.1; XP_022043008.1
Acipenser ruthenus	LOC117965185; LOC117422504; LOC117422503 LOC117401683; LOC117396711; LOC117396710	XP_034766114.1; XP_033893513.2; XP_033893512.1; XP_033858368.2; XP_033850746.1; XP_033850745.2
Amphiprion ocellaris	LOC111584976; LOC111584981; LOC111584980; LOC111584977	XP_035796893.1; XP_023150123.1; XP_023150115.1; XP_023150116.1; XP_023150121.1; XP_035796892.1; XP_023150117.1
Anabas testudineus	LOC113150921; LOC113150359; LOC113150357; LOC113151032; LOC113151031; LOC113151025; LOC113150512; LOC113150511; LOC113150506; LOC113150505; LOC113150496; LOC113150356; LOC113150355; LOC113150354; LOC113151081; LOC113151043; LOC113151040; LOC113150967; LOC113150509; LOC113150508; LOC113150507; LOC113150503; LOC113150502; LOC113150360; LOC113150353	XP_026199457.1; XP_026199458.1; XP_026198609.1; XP_026198608.1; XP_026198606.1; XP_026198607.1; XP_026199622.1; XP_026199621.1; XP_026199612.1; XP_026198845.1; XP_026198844.1; XP_026198843.1; XP_033182037.1; XP_026198837.1; XP_026198605.1; XP_026198604.1; XP_026198603.1; N/A (11)
Anarrhichthys ocellatus	LOC116400752; LOC116396188; LOC116396186; LOC116396185; LOC116396179; LOC116396120; LOC116378516; LOC116378017; LOC116378016	XP_031734625.1; XP_031726488.1; XP_031726486.1; XP_031726485.1; XP_031726472.1; XP_031726392.1; XP_031696264.1; N/A (2)
Archocentrus centrarchus	LOC115776052; LOC115776086; LOC115775888	XP_030579517.1; XP_030579516.1; XP_030579570.1; XP_030579258.1
Astatotilapia calliptera	LOC113033714; LOC113034237; LOC113032999; LOC113034238; LOC113034196	XP_026043554.1; XP_026043556.1; XP_026044423.1; XP_026042003.1; N/A (2)
Astyanax mexicanus	LOC103027322; LOC111191763; LOC111194308; LOC103027025	XP_007253908.2; XP_022523356.1; XP_022523353.1; XP_022533734.1; XP_022523255.1
Austrofundulus limnaeus	LOC106521840; LOC106521839; LOC106521842	XP_013870038.1; XP_013870037.1; N/A (1)
Betta splendens	LOC114870628; LOC114862698; LOC114862699; LOC114862486	XP_029031351.1; XP_029031362.1; XP_029031373.1; XP_029031382.1; XP_029019122.1; XP_029019123.1; XP_029019124.1; XP_029019125.1; XP_029019127.1; XP_029018660.1
Boleophthalmus pectinirostris	LOC110172491	XP_020794036.1
Carassius auratus	LOC113118046; LOC113067465; LOC113037668	XP_026142691.1; XP_026142692.1; XP_026095673.1; XP_026050760.1
Chanos chanos	LOC115827962; LOC115827961;	XP_030647716.1; XP_030647715.1;
Clupea harengus	LOC116220761; LOC105901666; LOC116217906; LOC105906341; LOC105906166; LOC105901667; LOC116222550	XP_031424776.1; XP_031424775.1; XP_031424777.1; XP_031424778.1; XP_012684608.2; XP_031432742.1; XP_031424779.1; XP_012689920.2; XP_031424780.1; XP_031431589.1; N/A (1)
Cottoperca gobio	LOC115013931; LOC115013914; LOC115013894; LOC115013596; LOC115013910	XP_029296358.1; XP_029296339.1; XP_029296316.1; XP_029295877.1; N/A (1)
Cyclopterus lumpus	LOC117736445; LOC117736998; LOC117736987; LOC117736986;	XP_034397685.1; XP_034397684.1; XP_034398593.1; XP_034398576.1; XP_034398574.1;

	LOC117735835; LOC117735834; LOC117735833; LOC117735832	XP_034396614.1; XP_034396612.1; XP_034396611.1; XP_034396610.1
Cynoglossus semilaevis	LOC107988277; LOC103398022; LOC103398021	XP_016886653.2; XP_008334723.1; XP_008334722.1
Cyprinodon variegatus	LOC107092866; LOC107091596; LOC107091586; LOC107087858; LOC107087312	XP_015243042.1; XP_015241089.1; XP_015241077.1; XP_015235140.1; XP_015234320.1
Cyprinus carpio	LOC109093544; LOC109075233; LOC109059965; LOC109048558 LOC109048500; LOC109095792; LOC109072820; LOC109054861	XP_018962823.1; XP_018946706.1; XP_018932680.1; XP_018921751.1; XP_018921710.1; N/A (3)
Danio rerio	LOC101882550; LOC795232; LOC564660; LOC564481 aep1; si:dkeyp-32g11.8; jac1; jac2; jac3; jac4	XP_021325134.1; XP_021329119.1; XP_017212453.1; NP_001373566.1; NP_001373496.1; NP_001013322.1; XP_001332452.2; XP_005166416.2; XP_021333376.1; XP_689401.2; XP_001920106.1; XP_001335438.1
Denticeps clupeoides	LOC114785644; LOC114785590	XP_028827979.1; XP_028827846.1
Echeneis naucrates	LOC115048490; LOC115048489	XP_029365835.1; XP_029365834.1
Electrophorus electricus	LOC113572950; LOC113572946; LOC118242386	XP_026858729.2; XP_026858721.2; N/A (1)
Epinephelus lanceolatus	LOC117253166; LOC117253049; LOC117253206; LOC117253205; LOC117253120; LOC117252549; LOC117252547; LOC117252363; LOC117252343	XP_033476433.1; XP_033476434.1; XP_033476276.1; XP_033476277.1; XP_033476485.1; XP_033476484.1; XP_033476370.1; XP_033475455.1; XP_033475453.1; XP_033475070.1; XP_033475041.1
Erpetoichthys calabaricus	LOC114660653; LOC114661331; LOC114657151; LOC114657149; LOC114657148; LOC114657147; LOC114657145	XP_028669333.1; XP_028669330.1; XP_028669326.1; XP_028669327.1; XP_028669323.1; XP_028669324.1; XP_028669322.1; XP_028669331.1; XP_028669332.1; XP_028669329.1; XP_028669325.1; XP_028670378.1; XP_028664731.1; XP_028664730.1; XP_028664729.1; XP_028664728.1; XP_028664726.1
Esox lucius	LOC105012483; LOC105014671; LOC105014670; LOC105009886	XP_010871658.1; XP_010871659.2; XP_019905734.1; XP_010875473.1; XP_010875472.2; NP_001291101.1
Etheostoma cragini	LOC117944414; LOC117944419; LOC117944417; LOC117944416; LOC117944415	XP_034727099.1; XP_034727100.1; XP_034727106.1; XP_034727104.1; XP_034727103.1; XP_034727101.1
Etheostoma spectabile	LOC116689688; LOC116689878; LOC116689689; LOC116689515; LOC116689372; LOC116689687	XP_032372170.1; XP_032372171.1; XP_032372421.1; XP_032372172.1; XP_032371965.1; XP_032371798.1; N/A (1)
Fundulus heteroclitus	LOC105921682; LOC105922588; LOC105922613; LOC105922611; LOC105922587; LOC118564905	XP_035999639.1; XP_035999640.1; XP_012713960.2; XP_036000698.1; XP_036000190.1; N/A (1)
Gadus morhua	LOC115548330; LOC115549398; LOC115550779	XP_030218725.1; XP_030220404.1; XP_030221930.1
Gouania willdenowi	LOC114470242; LOC114470241; LOC114469859; LOC114469797	XP_028314104.1; XP_028314103.1; XP_028313537.1; XP_028313410.1
Gymnodraco acuticeps	LOC117555386; LOC117555367; LOC117555398	XP_034086171.1; XP_034086172.1; XP_034086174.1; XP_034086175.1; XP_034086136.1; N/A (1)
Haplochromis burtoni	LOC102304550; LOC102304044; LOC102303736;	XP_005947701.1; XP_005947702.1; XP_005947699.1; XP_005947701.1; XP_005947702.1; XP_005947698.1
Hippocampus comes	LOC109524652; LOC109524648; LOC109524650 LOC109524649; LOC109524643; LOC109524642	XP_019740213.1; XP_019740214.1; XP_019740210.1; XP_019740209.1; XP_019740212.1; XP_019740211.1; XP_019740204.1; XP_019740203.1
Hippoglossus hippoglossus	LOC117768410; LOC117768415	XP_034452610.1; XP_034452611.1; XP_034452619.1
Hippoglossus stenolepis	LOC118115226; LOC118115225	XP_035022137.1; XP_035022136.1
Ictalurus punctatus	LOC108277208; LOC108256021; LOC108256022; LOC108281066; LOC108281062; LOC108254749; LOC108254737; LOC108277228	XP_017345216.1; XP_017345225.1; XP_017307974.1; XP_017307975.1; XP_017307974.1; XP_017352100.1; XP_017352097.1; XP_017305510.1; XP_017305499.1; N/A (1)
Kryptolebias marmoratus	LOC108242784; LOC108242761; LOC108242759; LOC108242786	XP_017283302.1; XP_017283303.1; XP_024864245.1; XP_017283277.2; XP_017283277.2; XP_024864245.1
Labrus bergylta	LOC110004881; LOC110004595; LOC110004882; LOC110003748; LOC110003723; LOC110002607; LOC110005408; LOC110003747	XP_029138746.1; XP_020516030.1; XP_029138670.1; XP_029138671.1; XP_020516031.2; XP_029138368.1; XP_020514928.1; XP_029138081.1; N/A (1)
Larimichthys crocea	LOC104935236; LOC104934802	XP_010749348.2; XP_010748853.3
Lates calcarifer	LOC108881648; LOC108881647; LOC108881649;	XP_018529232.1; XP_018529233.1; XP_018529229.1; XP_018529230.1;

	LOC108881646; LOC108881644; LOC108881593	XP_018529234.1; XP_018529228.1; XP_018529223.1; N/A (1)
Latimeria chalumnae	LOC102351619	XP_006007686.2
Lepisosteus oculatus	LOC102686741; LOC102686534; LOC102685725; LOC102685516; LOC102685315; LOC102685116; LOC102684513; LOC102683468; LOC102685926	XP_006630214.1; XP_006630213.1; XP_015201480.1; XP_006630210.1; XP_006630209.1; XP_015201478.1; XP_006628335.1; XP_015217114.1; N/A (1)
Mastacembelus armatus	LOC113139048; LOC113139041; LOC113138598; LOC113138597; LOC113138594	XP_026177782.1; XP_026177776.1; XP_026176934.1; XP_026176932.1; XP_026176928.1
Maylandia zebra	LOC101480981; LOC101486334; LOC101485557; LOC101480698; LOC101485828	XP_023008332.1; XP_023008333.1; XP_004555788.1; XP_004555785.1; XP_014263622.1; XP_023008331.1; XP_004555787.1
Monopterus albus	LOC109957452; LOC109957451; LOC109957450; LOC109957449	XP_020451000.1; XP_020450999.1; XP_020450998.1; XP_020450997.1
Morone saxatilis	LOC118326119; LOC118326022; LOC118325584; LOC118325583	XP_035514930.1; XP_035514798.1; XP_035514196.1; XP_035514195.1
Myripristis murdjan	LOC115365642; LOC115365590	XP_029916599.1; XP_029916535.1
Neolamprologus brichardi	LOC102791442; LOC102791148; LOC102790584; LOC102790864	XP_035766627.1; XP_035766593.1; XP_006797808.2; N/A (1)
Nothobranchius furzeri	LOC107395310; LOC107395311	XP_015830152.1; XP_015830153.1
Notolabrus celidotus	LOC117819157; LOC117819219; LOC117818360; LOC117819218; LOC117819388; LOC117819291; LOC117819186; LOC117818989; LOC117818538; LOC117818536 LOC117818361	XP_034548286.1; XP_034548287.1; XP_034548364.1; XP_034548366.1; XP_034548367.1; XP_034547094.1; XP_034547095.1; XP_034548362.1; XP_034548363.1; XP_034548598.1; XP_034548474.1; XP_034548320.1; XP_034548053.1; XP_034547367.1; XP_034547365.1; N/A (1)
Notothenia coriiceps	LOC104957312; LOC104943620	XP_010783235.1; XP_010767376.1
Oncorhynchus keta	LOC118393021; LOC118358151; LOC118357833	XP_035641051.1; XP_035591534.1; XP_035591045.1
Oncorhynchus kisutch	LOC109895078; LOC109873336; LOC109875722	XP_020344314.1; XP_031686132.1; XP_031674702.1; XP_031659281.1; XP_031659284.1
Oncorhynchus mykiss	LOC110492680; LOC110492679; LOC110492684; LOC110535045; LOC110525452; LOC110492682; LOC110492676; LOC110535046; LOC110492916; LOC110492683; LOC110492677	XP_021422808.1; XP_021422813.1; XP_021422805.1; XP_021422806.1; XP_021422817.1; XP_021475594.1; XP_021461251.1; XP_021422815.1; XP_021422803.1; N/A (4)
Oncorhynchus nerka	LOC115140663; LOC115139847; LOC115111420	XP_029534857.1; XP_029533533.1; XP_029493305.1
Oreochromis aureus	LOC116333913; LOC116316320; LOC116316319	XP_031613067.1; XP_031590732.1; XP_031590731.1
Oreochromis niloticus	LOC109204646; LOC102078591; LOC100690731; LOC112848328; LOC112848327; LOC109204627; LOC109204394; LOC106098185; LOC100712276; LOC100699724; LOC100699185; LOC102076794	XP_019221741.1; XP_019221742.1; XP_019221678.1; XP_019221679.1; XP_019221743.1; XP_013126388.2; XP_025767791.1; XP_025767789.1; XP_019221680.1; XP_025752615.1; XP_013126389.1; XP_019220826.1; XP_003460225.2; XP_003460223.2; N/A (1)
Oryzias latipes	LOC110017492; LOC101156471; LOC110017491; LOC105354800	XP_020569178.1; XP_004066450.1; XP_020569176.1; XP_011477929.1
Oryzias melastigma	LOC112148938; LOC112148433; LOC112141888; LOC112140920	XP_036069659.1; XP_036065765.1; XP_024120858.1; XP_024119711.1
Pangasianodon hypophthalmus	LOC113537151; LOC113537127	XP_026787297.1; XP_026787254.1
Paralichthys olivaceus	LOC109646373; LOC109623586	XP_019967672.1; XP_019933636.1
Parambassis ranga	LOC114442039; LOC114441490; LOC114442043; LOC114442041; LOC114441673; LOC114442042; LOC114441719; LOC114441718; LOC114441612	XP_028271100.1; XP_028271101.1; XP_028271102.1; XP_028270247.1; XP_028270248.1; XP_028270249.1; XP_028271107.1; XP_028271108.1; XP_028271103.1; XP_028271104.1; XP_028270505.1; XP_028270506.1; XP_028271105.1; XP_028270577.1; XP_028270576.1 XP_028270413.1

Paramormyrops kingsleyae	LOC111838587	XP_023657493.1
Perca flavescens	LOC114555804; LOC114556283; LOC114556282; LOC114556281; LOC114556279; LOC114556278; LOC114556277; LOC114556276; LOC114556275; LOC114556273; LOC114556272	XP_028434306.1; XP_028434994.1; XP_028434993.1; XP_028434992.1; XP_028434990.1; XP_028434989.1; XP_028434987.1; XP_028434986.1; XP_028434985.1; XP_028434984.1; XP_028434983.1
Periophthalmus magnuspinnatus	LOC117376833; LOC117376832	XP_033829269.1; XP_033829267.1
Poecilia formosa	LOC107833069; LOC107832875; LOC103152651; LOC103152639; LOC103152638; LOC103152637; LOC103131408	XP_016516744.1; XP_016516735.1; XP_007573269.2; XP_016516738.1; XP_007573254.1; XP_007573252.1; XP_007543104.1
Poecilia latipinna	LOC106964481; LOC106962433; LOC106936032; LOC106936031; LOC106936024; LOC106936023; LOC106936025	XP_014915517.1; XP_014912337.1; XP_014872294.1; XP_014872293.1; XP_014872285.1; XP_014872284.1; N/A (1)
Poecilia mexicana	LOC106929144; LOC106929143; LOC106929142; LOC106929132; LOC106929131; LOC106929128; LOC106913515	XP_014861318.1; XP_014861317.1; XP_014861316.1; XP_014861301.1; XP_014861300.1; XP_014861295.1; XP_014835615.1
Poecilia reticulata	LOC103470712; LOC103470711; LOC103470710; LOC108166572	XP_008417566.1; XP_008417564.1; XP_008417563.1; N/A (1)
Pseudochaenichthys georgianus	LOC117451939; LOC117451873	XP_033946207.1; XP_033946114.1
Pundamilia nyererei	LOC102196904; LOC102215630; LOC102215341	XP_005730650.1; XP_005730652.1; XP_005730718.1; XP_005730717.1
Pygocentrus nattereri	LOC108440524; LOC108440525; LOC108440519; LOC108440513; LOC108431588; LOC108416907; LOC108412640; LOC108412639	XP_017574907.1; XP_017574909.1; XP_017574901.1; XP_017574891.1; XP_017560326.1; XP_017545225.1; XP_017540238.1; XP_017540237.1
Salarias fasciatus	LOC115398569; LOC115397816; LOC115385734; LOC115385726	XP_029961263.1; XP_029960147.1; XP_029943660.1; XP_029943654.1
Salmo salar	nattl; LOC106585406; LOC106592833; LOC106598085; LOC106597996; LOC106585404; LOC106580260; LOC106566391; LOC106597753; LOC106590951; LOC106566392; LOC106566390; LOC106566389	NP_001134309.1; XP_014027037.1; XP_014027038.1; XP_014027036.1; XP_014027039.1; XP_014027040.1; XP_014039655.1; XP_014044638.1; XP_014044567.1; XP_014027033.1; XP_014016555.1; XP_013989840.1; N/A (5)
Salmo trutta	LOC115164743; LOC115199896; LOC115199582; LOC115195641; LOC115164744	XP_029573366.1; XP_029618292.1; XP_029617775.1; XP_029611579.1; N/A (1)
Salvelinus alpinus	LOC111974875; LOC111974874; LOC111953291	XP_023858706.1; XP_023858705.1; XP_023828243.1
Sander lucioperca	LOC116054306; LOC116054307; LOC118496505; LOC116054478; LOC116054465; LOC116054065; LOC116054064; LOC116054063; LOC116054062	XP_031161648.1; XP_031161649.1; XP_035852969.1; XP_031161650.1; XP_035852972.1; XP_035852974.1; XP_035852977.1; XP_035864307.1; XP_031161909.1; XP_031161888.1; XP_031161243.1; XP_031161242.1; XP_031161240.1; XP_031161239.1
Scleropages formosus	LOC114912550; LOC108923642; LOC108930950; LOC108930949	XP_029115652.1; XP_029115653.1; XP_018590053.1; XP_018590054.1; XP_018601972.1; XP_018601971.1
Scophthalmus maximus	LOC118314101; LOC118314103; LOC118314102; LOC118313943	XP_035496142.1; XP_035496145.1; XP_035496144.1; XP_035495834.1
Seriola dumerili	LOC111238558; LOC111238552; LOC111238550; LOC111238561; LOC111238559; LOC111238554	XP_022623850.1; XP_022623843.1; XP_022623842.1; XP_022623852.1; XP_022623851.1; XP_022623846.1
Seriola lalandi dorsalis	LOC111645714; LOC111645706; LOC111645703; LOC111645701	XP_023250786.1; XP_023250776.1; XP_023250770.1; XP_023250768.1
Sinocyclocheilus anshuiensis	LOC107685410; LOC107685411; LOC107703886; LOC107685416; LOC107697465; LOC107685415; LOC107685412; LOC107676233; LOC107675366	XP_016337469.1; XP_016337470.1; XP_016337473.1; XP_016362173.1; XP_016337476.1; N/A (5)
Sinocyclocheilus grahami	LOC107580454; LOC107572566; LOC107572132; LOC107571304; LOC107568250; LOC107568249; LOC107553807; LOC107553787; LOC107553785; LOC107574143;	XP_016121641.1; XP_016114476.1; XP_016114032.1; XP_016113107.1; XP_016109671.1; XP_016109670.1; XP_016091601.1; XP_016091579.1; XP_016091578.1; N/A (5)

	LOC107553768; LOC107553767; LOC107553766; LOC107553765	
Sinocyclocheilus rhinocerous	LOC107745953; LOC107750790; LOC107745964; LOC107745955; LOC107745954; LOC107732150; LOC107715051; LOC107750780; LOC107750779; LOC107735655; LOC107735617; LOC107729343	XP_016415472.1; XP_016421795.1; XP_016415481.1; XP_016415474.1; XP_016415473.1; XP_016398905.1; XP_016376580.1; N/A (5)
Sparus aurata	LOC115581715; LOC115582173; LOC115581714; LOC115581676; LOC115581675; LOC115581674; LOC115581671	XP_030272880.1; XP_030272881.1; XP_030273824.1; XP_030272879.1; XP_030272824.1; XP_030272823.1; XP_030272822.1; N/A (1)
Sphaeramia orbicularis	LOC115426097; LOC115425776; LOC115425773; LOC115425537	XP_029999915.1; XP_029999391.1; XP_029999388.1; XP_029999028.1
Stegastes partitus	LOC103375365; LOC103374718; LOC103368923; LOC103368914	XP_008303846.1; XP_008303071.1; XP_008295688.1; XP_008295674.1
Tachysurus fulvidraco	LOC113637608; LOC113637596; LOC113637595; LOC113637543	XP_026994141.1; XP_026994116.1; XP_026994115.1; N/A (1)
Takifugu rubripes	LOC101080080; LOC101061072	XP_029685569.1; XP_011613836.1
Thalassophryne amazonica	LOC117507937; LOC117507935; LOC117506200; LOC117502539; LOC117511181; LOC117511179; LOC117511178; LOC117511154; LOC117509998; LOC117509997; LOC117509883; LOC117509882; LOC117509876; LOC117509873; LOC117509872; LOC117509871; LOC117509869; LOC117509868; LOC117509867; LOC117509866; LOC117509865; LOC117507942; LOC117507938; LOC117507762; LOC117507040; LOC117506750	XP_034023579.1; XP_034023580.1; XP_034023576.1; XP_034023577.1; XP_034021587.1; XP_034021588.1; XP_034017472.1; XP_034017473.1; XP_034027058.1; XP_034027056.1; XP_034027055.1; XP_034027023.1; XP_034025496.1; XP_034025495.1; XP_034025402.1; XP_034025400.1; XP_034025396.1; XP_034025394.1; XP_034025393.1; XP_034025391.1; XP_034025390.1; XP_034025389.1; XP_034025388.1; XP_034025387.1; XP_034025386.1; XP_034023585.1; XP_034023581.1; XP_034023462.1; XP_034022621.1; XP_034022222.1
Trematomus bernacchii	LOC117499337; LOC117473203; LOC117472357	XP_034007571.1; XP_033974611.1; XP_033973395.1
Xiphophorus couchianus	LOC114154796; LOC114154797	XP_027890011.1; XP_027890013.1
Xiphophorus hellerii	LOC116729175; LOC116729620; LOC116729293; LOC116729243; LOC116729623	XP_032433435.1; XP_032433436.1; XP_032434181.1; XP_032433613.1; XP_032433527.1; N/A (1)
Xiphophorus maculatus	LOC102218571; LOC102235791; LOC102223347; LOC102218316	XP_023198850.1; XP_023198851.1; XP_023199097.1; XP_023199098.1; XP_023199805.1
	**UNIPROT**	
Anabarilius grahami (Barilius grahami)	DPX16_2196; DPX16_2659	A0A3N0Z5Q4-1; A0A3N0Y4R9-1
Channa argus	EXN66_Car011057; EXN66_Car011056; EXN66_Car011033; EXN66_Car011054; EXN66_Car011055; EXN66_Car011053;	A0A6G1PYG8-1; A0A6G1PYG9-1; A0A6G1PYN7-1; A0A6G1PZF2-1; A0A6G1PYT2-1; A0A6G1PYQ5-1
Collichthys lucidus (*Sciaena lucida*)	D9C73_012990; D9C73_013011; D9C73_013010; D9C73_012991	A0A4U5USR6-1; A0A4U5UST4-1; A0A4U5USP8-1; A0A4U5USV2-1
Ictalurus furcatus (*Pimelodus furcatus*)	NATTL	E3TBY7-1
Labeo rohita (*Cyprinus rohita*)	ROHU_028553; ROHU_025781; ROHU_028551; ROHU_028552; ROHU_007491; ROHU_028549; ROHU_028879; ROHU_003160; ROHU_026098	A0A498M4F8-1; A0A498MQR0-1; A0A498M2J8-1; A0A498M5V3-1; A0A498MFS9; A0A498M1M4-1; A0A498M4E6-1; A0A498NW31; A0A498MBY3
Lethenteron camtschaticum (*Lampetra japonica*)	N/A	K7WEH5-1
Liparis tanakae	NATT4_2; NATT3; NATT4_0; NATT4_1	A0A4Z2FCX0-1; A0A4Z2FCH6-1; A0A4Z2FDY0-1; A0A4Z2FCV9-1
Plotosus lineatus (*Silurus lineatus*)	pltx-II; pltx-I	F2ZAL6-1; F2ZAL5-1
Takifugu flavidus	D4764_05G0009030; D4764_05G0009020	A0A5C6N0M3-1; A0A5C6N045

Thalassophryne nattereri	NATTERIN 1, NATTERIN 2, NATTERIN 3, NATTERIN 4,	Q66S25-1; Q66S21; Q66S17-1; Q66S13-1
**Literature**		
Eudontomyzon morii (Lampetra morii)	N/A	N/A
Plotosus canius	N/A	N/A
Trachinotus ovatus	N/A	N/A

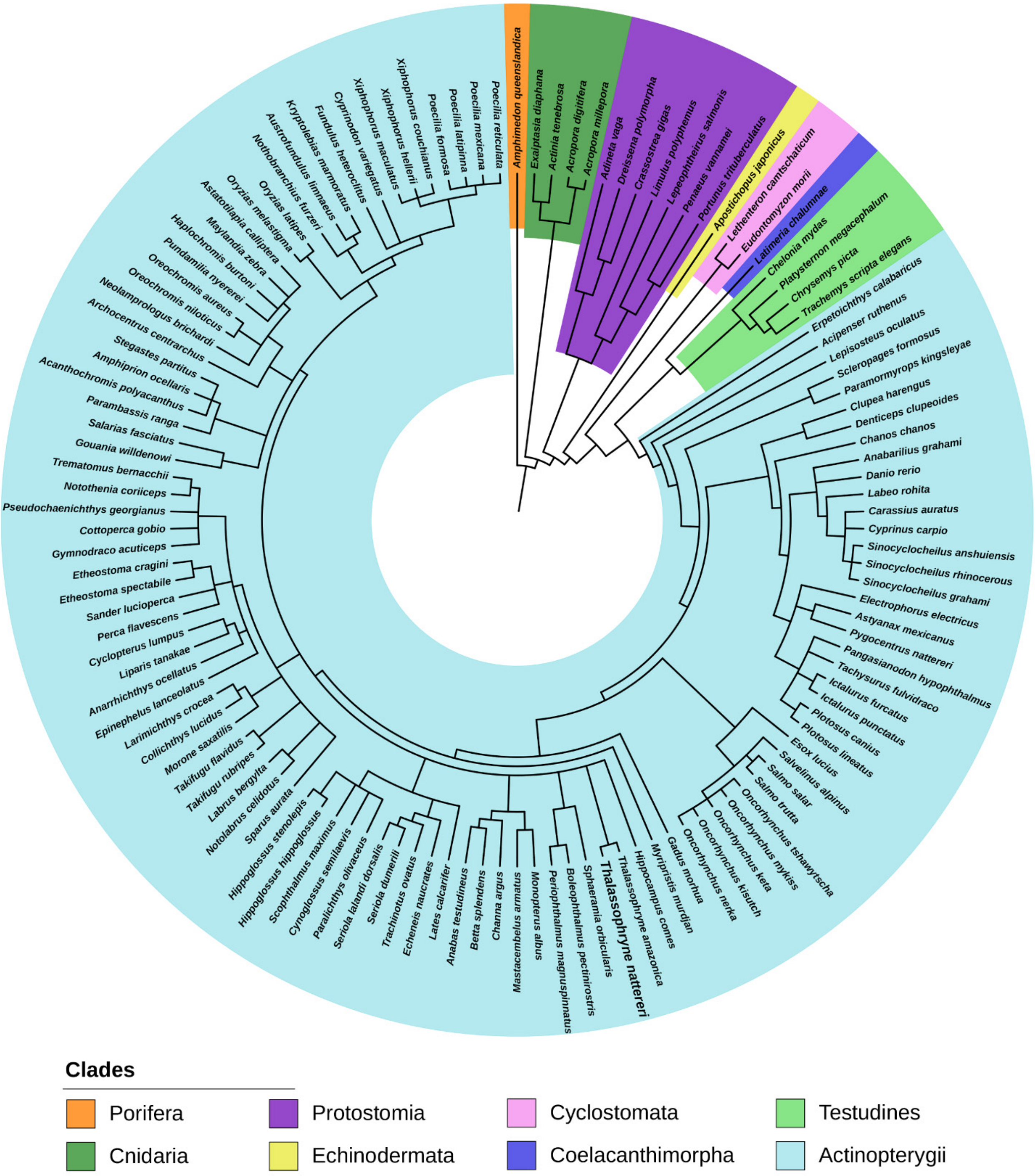

Figure S1. Phylogenetic tree generated using the software PhyloT to represent the aquatic species included in the group of Natterin-like proteins. There are representatives from Porifera to ray-finned fish (Actinopterygii).

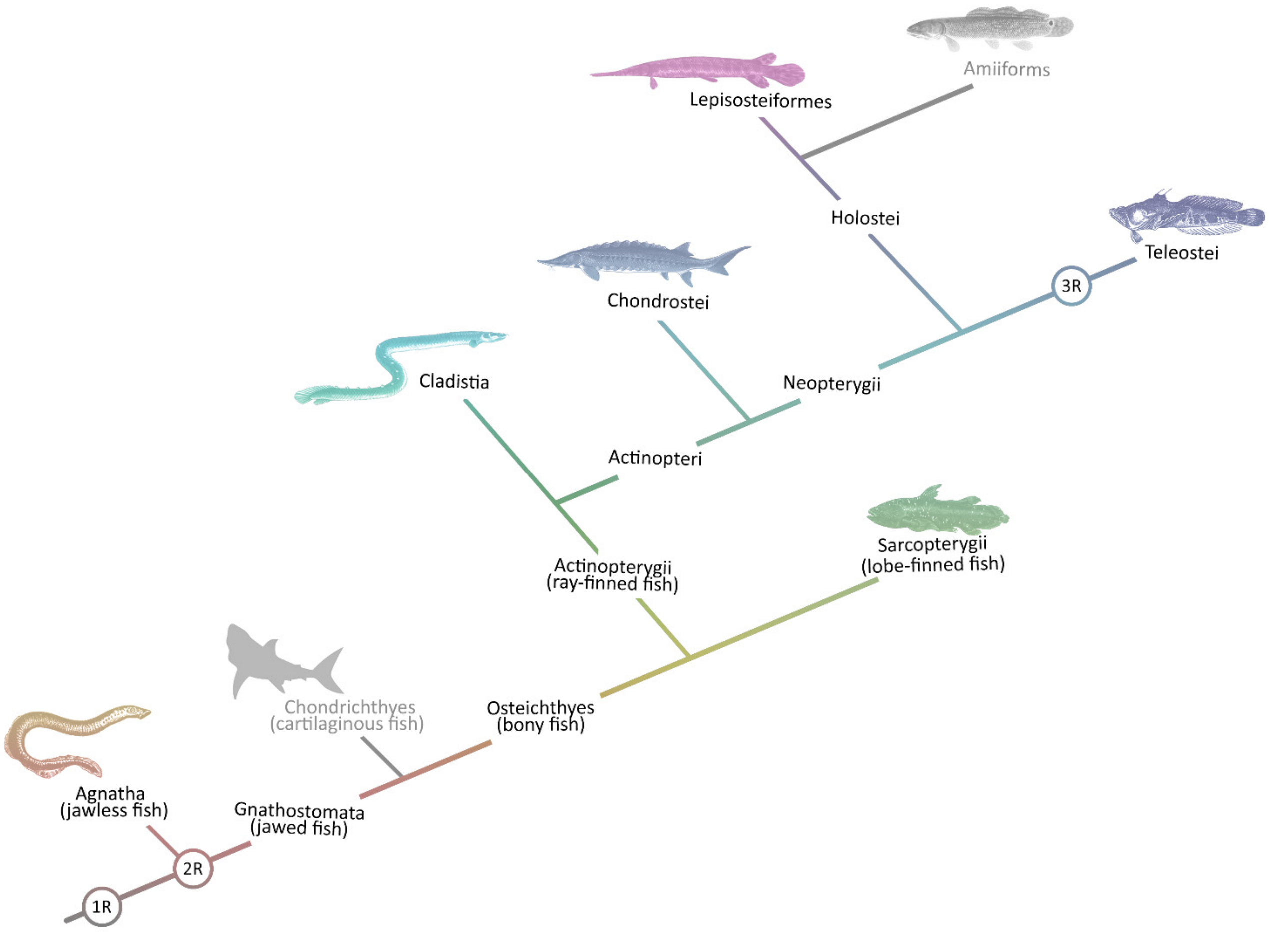

Figure S2. The general classification of fishes, a paraphyletic assemblage including the Agnatha, Chondrichthyes, Sarcopterygii, and Actinopterygii. Groups uncolored do not have representatives included in the group of Natterin-like proteins. The three rounds of whole-genome duplication (WGD) events are pointed to in the cladogram. .
