# Supplementary figures and images for: The Natterin Proteins Diversity: A Review on Phylogeny, Structure, and Immune Function
Source: Toxins (Basel). 2021 Jul 31;13(8):538. doi: 10.3390/toxins13080538 (PMC8402412; doi:10.3390/toxins13080538)

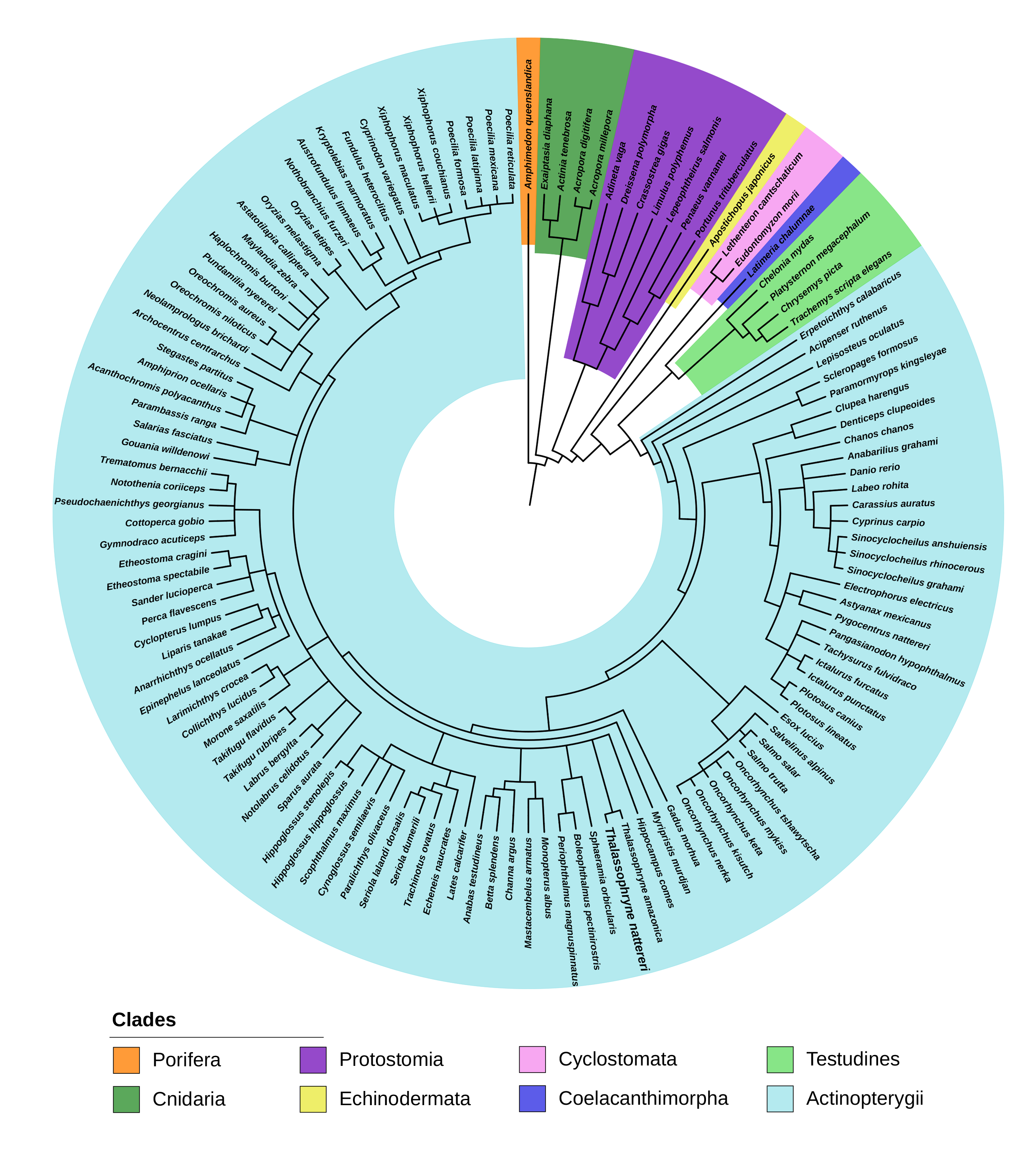

Supplement: Supplementary file 1 [file toxins-13-00538-s001.zip › Figure S1.tif]

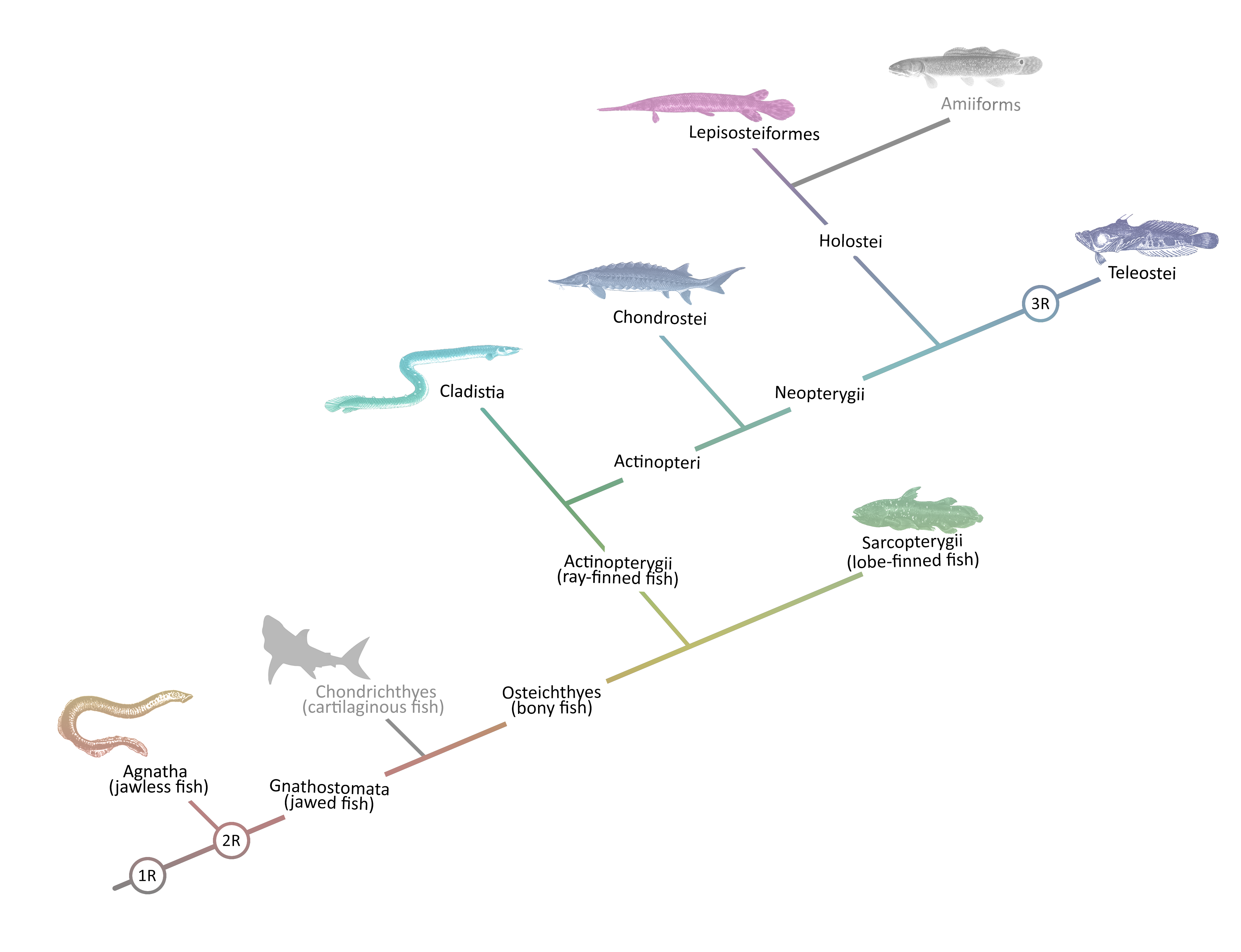

Supplement: Supplementary file 1 [file toxins-13-00538-s001.zip › Figure S2.tif]
